# Amino acid sequence encodes protein abundance shaped by protein stability at reduced synthesis cost

**DOI:** 10.1002/pro.5239

**Published:** 2024-12-12

**Authors:** Filip Buric, Sandra Viknander, Xiaozhi Fu, Oliver Lemke, Oriol Gracia Carmona, Jan Zrimec, Lukasz Szyrwiel, Michael Mülleder, Markus Ralser, Aleksej Zelezniak

**Affiliations:** ^1^ Department of Biology and Biological Engineering Chalmers University of Technology Gothenburg Sweden; ^2^ Department of Biochemistry Charité – Universitätsmedizin Berlin Berlin Germany; ^3^ Randall Centre for Cell & Molecular Biophysics King's College London London UK; ^4^ Institute of Structural and Molecular Biology University College London London UK; ^5^ Department of Biotechnology and Systems Biology National Institute of Biology Ljubljana Slovenia; ^6^ Core Facility High Throughput Mass Spectrometry Charité – Universitätsmedizin Berlin Berlin Germany; ^7^ Institute of Biotechnology, Life Sciences Centre Vilnius University Vilnius Lithuania

**Keywords:** deep learning, explainable machine learning, language models, molecular dynamics, protein engineering, protein expression, protein sequence, protein stability, proteome

## Abstract

Understanding what drives protein abundance is essential to biology, medicine, and biotechnology. Driven by evolutionary selection, an amino acid sequence is tailored to meet the required abundance of a proteome, underscoring the intricate relationship between sequence and functional demand. Yet, the specific role of amino acid sequences in determining proteome abundance remains elusive. Here we show that the amino acid sequence alone encodes over half of protein abundance variation across all domains of life, ranging from bacteria to mouse and human. With an attempt to go beyond predictions, we trained a manageable‐size Transformer model to interpret latent factors predictive of protein abundances. Intuitively, the model's attention focused on the protein's structural features linked to stability and metabolic costs related to protein synthesis. To probe these relationships, we introduce MGEM (Mutation Guided by an Embedded Manifold), a methodology for guiding protein abundance through sequence modifications. We find that mutations which increase predicted abundance have significantly altered protein polarity and hydrophobicity, underscoring a connection between protein structural features and abundance. Through molecular dynamics simulations we revealed that abundance‐enhancing mutations possibly contribute to protein thermostability by increasing rigidity, which occurs at a lower synthesis cost.

## 1 INTRODUCTION

The intricate interplay between protein synthesis and degradation defines intracellular protein levels, with implications for therapeutic strategies, protein and cellular engineering. The complex regulation of protein homeostasis suggests that multiple factors contribute to the overall proteome makeup, with the evolutionarily encoded sequence potentially playing a pivotal role in proteome composition. For instance, protein synthesis is strongly regulated at the initiation step (Laursen et al. 2005; Merrick and Pavitt 2018; Verma et al. 2019), whose rate varies broadly between mRNAs, depending not only on the transcript sequence features (Vogel et al. 2010; Zur and Tuller 2013) but also on the amino acids at the N‐terminal (Goodman et al. 2013; Zhao et al. 2019). In bacteria, the amino acid composition of the C‐terminal is a strong determinant of protein degradation rates, explaining a wide range of protein abundances (Correa Marrero and Barrio‐Hernandez 2021; Weber et al. 2020). These, along with the multiple mechanisms of post‐translational regulation (Müller 2018; Tokmakov et al. 2012), suggest that this rather tight regulation occurs at the degradation level and is encoded, at least partially, in the amino acid sequence. Empirically, amino acid composition and sequence features were seen to correlate with protein abundance (Cascarina and Ross 2018; Riba et al. 2019; van den Berg et al. 2012), protein sequence redesign led to an order of magnitude higher increase of protein abundance compared with codon optimization (van den Berg et al. 2014), transcending mere codon composition influences on protein abundance (Ikemura 1985). While the importance of protein sequence in determining abundance is recognized, the quantitative relationship between sequence and abundance remains elusive, as does the link between the evolutionary mechanisms that underlie this relationship.

On a broader scale, proteins situated as central players in cellular processes or as critical nodes in interaction networks often exhibit higher abundances (Jeong et al. 2001). Evolutionarily, these highly abundant proteins face stringent constraints, evolving at a slower pace due to their potential large‐scale impact on cellular fitness (Pál et al. 2006; Zhang and Yang 2015). Remarkably, the conservation of steady‐state protein abundances spans diverse evolutionary lineages, ranging from bacteria to humans (Laurent et al. 2010; Schrimpf et al. 2009; Tuller et al. 2010a). Theoretical models suggest that increasing protein abundance slows evolution due to reduced fitness, with the least stable proteins adapting the fastest (Agozzino and Dill 2018). Yet, under strong selection, proteins can evolve faster by adopting mutations that enhance stability and folding (Zheng et al. 2020). Experimental evidence also suggests that a protein's capacity to evolve is enhanced by the mutational robustness conferred by extra stability (Bloom et al. 2006; Bloom et al. 2007; Youssef et al. 2022), meaning that protein stability increases evolvability by allowing it to accept a broader range of beneficial mutations while still folding to its native structure. Thermostability gains of highly expressed orthologs are often accompanied by a more negative ΔG of folding, indicating that highly expressed proteins are often more thermostable (Luzuriaga‐Neira et al. 2023), as often explained by the so‐called misfolding avoidance hypothesis (MAH), because stable proteins are evolutionarily designed to tolerate translational errors (Drummond et al. 2005; Drummond and Wilke 2008; Leuenberger et al. 2017). On the contrary, several empirical studies revealed no substantial correlation between protein stability and protein abundance (Plata and Vitkup 2018; Usmanova et al. 2021). Likewise, the overall cost (per protein) of translation‐induced misfolding is low compared to the metabolic cost of synthesis (Nisthal et al. 2019; Yang et al. 2010), suggesting that MAH does not explain why highly abundant proteins evolve slower (Usmanova et al. 2021). On the other hand, cells may have fine‐tuned protein sequences to balance their functional importance with the metabolic costs they incur, reflecting an optimisation between functional necessity and energy efficiency (Akashi and Gojobori 2002; Cherry 2010; Gout et al. 2010). Given the intricate interplay of evolutionary constraints, protein stability, abundance, and protein synthesis metabolic cost, it remains unclear how cells evolved their sequences to strike an optimal balance between functional demands of proteome and cellular fitness associated with the synthesis and maintenance of protein abundance.

In this study, we explore the relationship between a protein's amino acid sequence and its abundance by asking: “How much does the protein *sequence* (as opposed to amino acid composition) predict protein abundance?” Using a large protein language Transformer (Lin et al. 2023), we showed that >50% of protein abundance variation can be predicted solely from the amino acid sequence, as shown in at least 38 species from all domains of life, including *Homo sapiens*. To understand details, we focused on the model organism *Saccharomyces cerevisiae*, and trained an interpretable deep neural network Transformer architecture (*R* ^2^ test = 56%) to predict protein abundance. Delving into the neural network's self‐attention mechanism with post hoc analyses to understand which protein sequence features predict their abundances, we found that the network indirectly identified multiple physicochemical features related to protein's structural properties and the overall metabolic features, such as synthesis costs, which the model pays attention to when predicting abundance. We then introduced MGEM (Mutation Guided by an Embedded Manifold) to probe sequence space. Mutations that increase predicted abundance using only Transformer‐derived positional residue importance values notably affected protein polarity and hydrophobicity, hinting at a stability‐abundance connection. Molecular dynamics simulations gave further evidence for the enhanced rigidity of abundance‐increasing mutants, a phenotype pronounced for thermo‐stabilizing mutations (Yu et al. 2017; Yu and Huang 2014). Importantly, we found that mutants with increased abundance had lower amino acid synthesis metabolic costs than their native versions, underscoring the fitness benefits of abundant proteins. Our results show that besides the amino acid composition, the sequence is a crucial factor predicting intracellular protein levels. Based on the factors we identified, this is conceivably achieved in part potentially by protein stabilization (through the increase of rigidity) and by cost‐effective amino acid substitutions, providing evolutionary benefits by reducing the metabolic costs of protein synthesis.

## 2 RESULTS

### 2.1 The amino acid sequence is generally predictive of protein abundance

**FIGURE 1:**
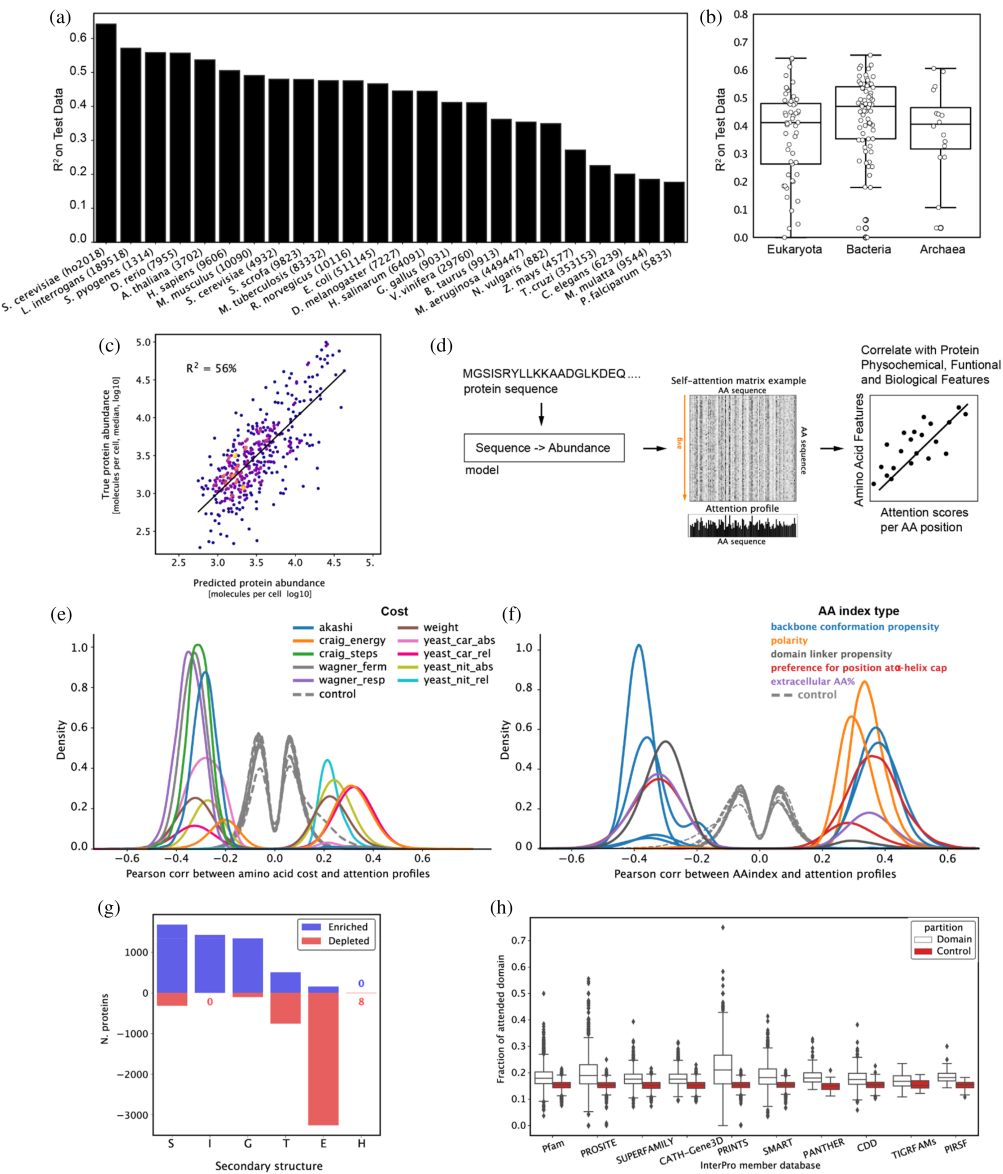
The amino acid sequence is predictive of protein abundance. (a) Sequence‐to‐abundance predictive performance on a hold‐out test set of large protein language models (LLMs) (Lin et al. 2023), using species with at least four proteome datasets from the PaxDb (Huang et al. 2023). (b) Overall predictive performance of fine‐tuned LLM Transformers using all datasets from the PaxDb. (c) Transformer performance on a hold‐out test set trained on the yeast dataset (Ho et al. 2018), colored by density. (d) A protein sequence is passed through the model to extract attention matrices from all layers. A Transformer attention matrix example and derived attention profile for a protein sequence. Attention matrices consist of directional association weights between pairs of residues, normalized as a percentage. The profiles were obtained by averaging along the “attends‐to” axis, as the “attended‐by” variation is generally more informative, resulting in one‐dimensional attention profiles that are then correlated to multiple protein features. (e) Attention profiles correlate with amino acid metabolic costs (see also Table S1 for full description). Shown are distributions across all sequences of maximum (absolute) Pearson correlations of any attention profile with *p*‐value <1e‐5, as well as a random control (gray, dashed) consisting of correlations produced the same way for shuffled versions of the same sequences. (f) Attention profiles correlate with 10 non‐redundant AAindex variables (colored by index type), showing that profiles capture information pertaining to backbone conformation, physicochemical properties, domain linkage, and secondary structure. While some AAindex types correlate with attention profiles both positively and negatively (e.g., backbone conformation), individual AAindex variables within these types are overall either positively or negatively correlated. The categories shown span AAindex variables that are both positively and negatively correlated with attention. Shown is also a random control (gray, dashed) consisting of correlations produced the same way for shuffled versions of the same sequences. As the mean abs correlation threshold (0.3) was removed for these, the plot shows the distributions for all 18 initial AAindex variables. (g) Proteins are split into two subpopulations of sequences with high attention values (*z*‐score >1) that are either enriched in turns and helices (S, I, G, and T in DSSP notation) and, to a lesser extent, extended strand (E), or largely depleted in extended strand (E) and turn (T), as assessed with one‐sided hypergeometric tests (p‐value <0.05). (h) Overlap of attention patterns with protein domains from the yeast InterPro database, grouped by member databases. The attention coverage of domains (fraction overlapping with attention profiles) is significantly higher than the control for 10 out of 12 member databases (Wilcoxon two‐sided signed‐rank test, *p*‐value <0.05), with the highest coverage in PRINTS and PROSITE.

Cellular protein levels are determined by the balance between multiple processes (Ho et al. 2018; Vogel and Marcotte 2012), but steady‐state abundances may be roughly approximated by the interplay between protein synthesis (involving transcriptionally related processes) and degradation, which can be exemplified by a simple model that incorporates the two key proxy factors: protein translation efficiency (Weinberg et al. 2016) (ribosome density normalized by transcript abundance) and protein half‐life (Christiano et al. 2014). A random forest model we trained on these two factors explains a relatively large proportion (*R* ^2^ test = 36%) of protein abundance variation, with 65% model contribution from translation efficiency and 35% from protein half‐life, respectively (Figure S1) (section 4.1). While it is evident that a protein's primary structure, its amino acid sequence, is related to protein synthesis and degradation, it is unclear to what extent the information about protein levels is encoded in the sequence. Thus, to investigate the relationship between amino acid sequence and protein abundance, we used a compendium of protein abundance estimates from PaxDb (Huang et al. 2023) of over 800 experimental studies across 136 species representing all domains of life, ranging from bacteria to humans. Namely, for each organism, we formulated a regression problem by utilizing protein sequences to model intracellular protein levels, by training a neural network using sequence representations derived from a pretrained large protein language model (Lin et al. 2023) (section 4.2). The predictive performance on independent test data (Figure 1a), using only an amino acid sequence as input, measured by *R* ^2^ overall, predicts 44% (median) of abundance variation across all domains of life (Figure 1b), including human tissues (Figures S2–S4), suggesting that the amino acid sequence encodes protein abundance.

We next attempted to look deeper to develop an interpretable model, as models derived from deep neural networks are often difficult to interpret (Savage 2022). Although protein sequence representations, so‐called embeddings, including sequence representations learned from structural models (Jumper et al. 2021), are useful for multiple tasks in protein science (Johnson et al. 2023; Kroll et al. 2023; Littmann et al. 2021), such vectorised protein sequence representations have been shown to have limited generalization to all protein functions and properties (Hu et al. 2022; Johnson et al. 2023), making it especially difficult to use for all‐purpose interpretation, that is, abundance prediction. Thus, in our case, to increase interpretability, we utilized a relatively small Transformer model trained entirely from scratch to obtain a direct map of sequence‐to‐protein abundances, as opposed to using pre‐trained large protein language models (Brandes et al. 2022; Ferruz et al. 2022; Madani et al. 2023; Rives et al. 2021). By training the model from scratch in a regression setting (section 4.3), we ensured that our model learned relevant sequence representations only aligned to protein abundance, thus easing further interpretation. To learn from the sequence, we chose Transformer with its multi‐head attention architecture (Devlin et al. 2018; Rao et al. 2019), which allows for some transparency in weighing the contributions of amino acid residues on protein levels and can provide insights into the most relevant sequence features the model uses (Rao et al. 2019; Rao et al. 2020; Vig et al. 2020) to make predictions about protein abundances, using an intrinsic attention mechanism (Vaswani et al. 2017). As for data, independently of the PaxDb dataset (Huang et al. 2023), we used a curated compendium of 21 experimental systematic studies employing mass spectrometry and microscopy techniques to estimate absolute protein abundances (copy numbers per cell) of over 5000 proteins in *S. cerevisiae* grown predominantly in the exponential phases across multiple conditions, essentially capturing known yeast proteome variation (Ho et al. 2018). Due to deep learning's need for extensive training data and the yeast dataset's limited size, we used repeated measurements (up to 21 sequence copies from all experiments in the dataset) to account for inter‐experimental variation (equivalent to regression with replicates). Our augmented dataset included 199,206 training examples, with 10% of sequences uniquely (and randomly) chosen (the same sequence is only in one data split) for validation during model training and 10% for a hold‐out test during the final model evaluation (section 4.3). Similarly, as with the protein language model, by training the smaller Transformer model from scratch, we found that the model predicts 56% of protein abundance variation (*R* ^2^ test = 56% on a holdout test set, RMSE = 14,303 [molecules per cell] corresponding to <1 of this set's standard deviation) using only an amino acid sequence as input (Figure 1c, again supporting that the sequence predominantly encodes protein abundance). In contrast, the model predictions failed when performing a randomization control with shuffled versions of the same test set sequences (*R* ^2^ = −73%, Figure S5), confirming that the model relies on residue interdependencies in a sequence rather than simply learning amino acid frequencies when predicting protein levels, and is thus complementary to composition‐based partial predictors. Further support of the network's ability to pick up information encoded in the sequence contrasts the above result with composition as a predictor of abundance. A multiple linear regression model using amino acid frequencies had an *R* ^2^ = 21% on the same test set. Indeed, the amino acid frequency varies only slightly across abundance deciles (Figure S6d).

### 2.2 The attention mechanism connects sequence and structural features to protein abundance

By focusing on the model organism *S. cerevisiae*, for which a high‐quality protein copy numbers dataset spanning 21 experiments was available (Ho et al. 2018), we next attempted to identify abundance‐related links to various physicochemical, biochemical, and functional protein features using the attention values derived from yeast protein sequences (Figure 1d). We extracted the attention weights of each input sequence. We obtained one‐dimensional per‐residue attention profiles, reflecting the average percentage of attention each residue receives from all others in the sequence when making the corresponding abundance prediction (see Figure S7 and section 4.4).

To examine the determinants of protein abundance, we first correlated attention profiles with amino acid metabolic costs (Barton et al. 2010) (section 4.5), as amino acid synthesis cost is known to be a determinant of protein abundance (Akashi and Gojobori 2002; Raiford et al. 2008; Swire 2007; Wagner 2005). The strongest correlations were found between attention profiles and the energetic cost of amino acids (*craig_energy*) (Craig and Weber 1998) averaged over all proteins (mean Pearson's *r* = 0.32, BH adj. *p*‐value <1e‐5). Conversely, anticorrelations were observed with synthetic cost under both respiratory and fermentative growth (*wagner_resp*, *wagner_ferm*, respectively) (Wagner 2005) as well as the number of synthesis steps (*craig_steps*) (Craig and Weber 1998) (mean Pearson's *r* = −0.35, −0.33, and −0.31, respectively, BH adj. *p*‐value <1e‐5). Additionally, some of the systemic costs introduced by Barton et al. (2010) using genome‐scale flux balance analysis calculations (Orth et al. 2010) showed positive and negative correlations with attention, such as the impact of the relative change of the amino acid requirement on the minimal intake of glucose (*yeast_car_rel*, mean Pearson's *r* = 0.32 over 1855 proteins and −0.33 over 705 proteins, BH adj. *p*‐value <1e‐5) and the absolute change of the amino acid requirement on the minimal intake of ammonium (*yeast_nit_abs*, mean Pearson's *r* = 0.25 over 1833 proteins and −0.28 over 1165 proteins, BH adj. *p*‐value <1e‐5, Figure 1e and Table S1). A negative correlation with synthesis cost implies that the model assigns more weight to “cheaply” synthesized amino acids. In contrast, a positive correlation with energy cost implies paying attention to more energy‐rich amino acids when predicting protein abundance. As random control, we performed the same procedure for shuffled versions of the same sequences, which yielded minuscule correlations, once again highlighting that attention captures positional information (Figure 1e). We stress that the correlations reported here do not directly link cost values to the predicted abundance but rather underline the relevant latent features learned from protein sequence that the model picked up intrinsically when mapping sequence to protein levels.

Based on our observation that amino acid frequency remains relatively constant across the entire dynamic range of protein abundances (Figure S6d), we did not expect to find specific single amino acids that would determine abundances. Instead, we hypothesized that the neural network would capture higher‐order interactions important for structural and functional protein features. Thus, we correlated attention profiles with a subset of 18 non‐redundant AAindex values representing various physicochemical and biochemical protein properties (Kawashima and Kanehisa 2000) (see section 4.6). We identified significant correlations with measures of backbone *conformation propensity* (both positively and negatively correlated indices, with the strongest mean correlations being 0.38 and −0.38, respectively, BH adj. *p*‐value <1e‐5), *preference for position at α‐helix cap* (both positively and negatively correlated indices, with the strongest mean correlations per sequence being 0.37 and −0.33, respectively, BH adj. *p*‐value <1e‐5), *polarity* (highest mean correlation = 0.35, BH adj. *p*‐value <1e‐5), *domain linker propensity* (mean correlation = −0.31, BH adj. *p*‐value <1e‐5), and *the composition of extracellular domains seen in membrane proteins* (two protein subpopulations, one with mean correlation = 0.36, the other with mean anticorrelation = −0.33, BH adj. *p*‐value <1e‐5) (Figure 1f and see Tables S2 and S3 for a detailed description). Physicochemical properties of amino acids, such as polarity, have been shown to affect translation speed (Riba et al. 2019) and protein stability (Panja et al. 2020; Tsuboyama et al. 2023). As opposed to random control (Figure 1f), the identified correlations with backbone conformation and preference for α‐helix cap indicators suggest a link to secondary structure. In contrast, the correlation with domain linker propensity points to the model having learned the boundaries of domain separation to some extent.

Next, we assessed the connection between secondary structure and attention profiles by analyzing the enrichment of per‐residue DSSP annotations (Kabsch and Sander 1983; Touw et al. 2015) in high‐attention positions using AlphaFold2‐generated (Jumper et al. 2021) structures for 4745 yeast proteins. We counted the annotations at positions with attention profile *z*‐scores >1 and compared them to background annotation counts across all proteins (using one‐sided hypergeometric tests for enrichment and depletion, *p*‐value <0.05) (section 4.7). The results showed that attention values were enriched in turns and helices (S, I, G, and T in DSSP notation) but depleted in extended strands (E) for most proteins (3254 proteins) (Figure 1g). For turns (T), the protein subpopulations were more evenly split, with this structure enriched in 505 proteins and depleted in 754 proteins. These findings suggest that helical structures may be implicated in protein abundance, while the contribution of turns and sheets towards the model prediction may be more complex.

As structural properties imply function, we also investigated whether abundance‐driven attention specifically focuses on any functional regions of protein sequences. We examined the extent to which the attention patterns cover the domains from the *S. cerevisiae* InterPro (Blum et al. 2021) database. To allow for comparison with controls, we focused only on domains with a length less than half of the protein sequence, analyzing a total of 18,000 domains (section 4.8). For 10 out of 12 member databases, domains were significantly more covered by high attention than random regions of the same length (Wilcoxon two‐sided signed‐rank test, adj. *p*‐value <0.05) (Figure 1h). The results are particularly striking as our Transformer model was trained from scratch, not pre‐trained on domains as in the study by Rao et al. (2019). We next performed a GO enrichment analysis on proteins with well‐covered domains (chosen as at least 30% domain length overlapping with attention patterns, well above the random control), a total of 832 domains in 517 proteins (section 4.9). From the enriched terms, GO‐slim terms were produced for summarization (Table S4). The enriched (Hypergeometric test, adj. *p*‐value <0.05) biological processes are diverse and, among others, include translation, protein folding, modification, and metabolic processes; the molecular functions include cytoskeletal protein binding, unfolded protein binding, DNA and RNA binding, transmembrane transporter activity and others. This variety points at widespread domain patterns to which the model attends across different protein classes rather than specific functional motifs, which hints at the role of sequence across the entire proteome. On the technical side of the attention mechanism itself, it is interesting to note that domains were predominantly captured by a single (and deeper) network layer (Figure S8).

### 2.3 Navigating the sequence space to control protein abundance

**FIGURE 2:**
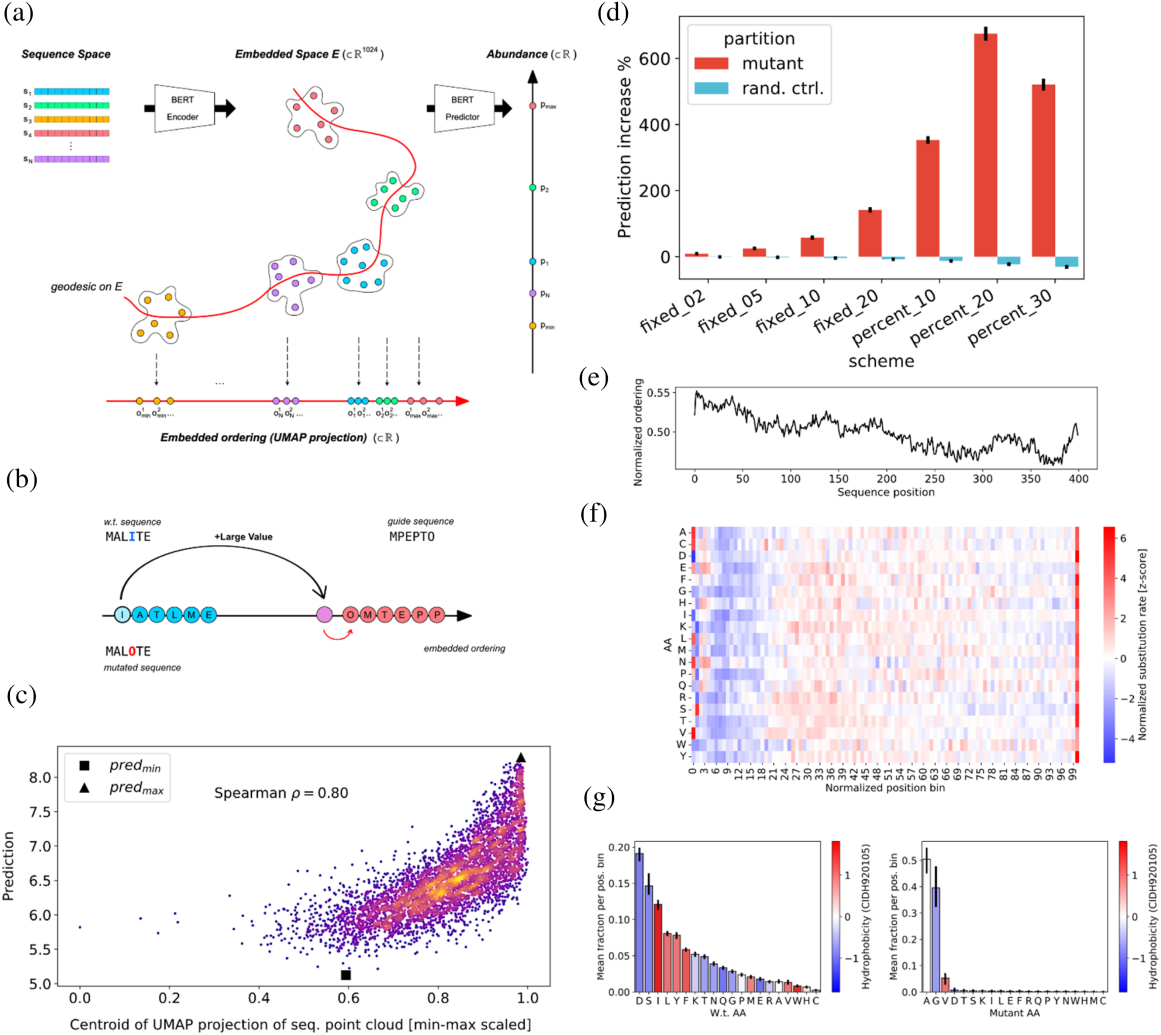
Navigating the sequence space to control protein abundance through guided mutation. (a) Conceptual illustration showing the posited structure of the Transformer encoder embedded space and the embedded ordering construction that supports our guided mutation procedure. The encoder maps each residue in a sequence to a high‐dimensional point in the embedded space *E* and sequences thus appear as point clouds. From a point cloud, a thin feedforward predictor yields an abundance prediction. The embedded space is posited to be structured in such a way as to allow a “traversal” of the point clouds, on a path or *geodesic* between all points (curved red line) connecting the points that are part of the lowest abundance sequences to the highest, in an increasing order of predicted values. This path in high‐dimensional space is approximated with a parametric UMAP projection from the embedded space *E* to a single dimension, thus giving a simple linear ranking (or ordering) *o*_*i*_^*j*^ for each residue *j*, in each sequence *i*. This ranking serves to indicate the global weight of a given residue towards the final prediction, compared with all other residues across all sequences. (b) Simplified illustration of MGEM (mutation guided by embedded manifold) procedure, which takes advantage of the global embedded order value (“importance”) obtained for each residue, across all sequences. The residues with the lowest order value in a sequence are selected for substitution (the “I” residue at position 4 in the illustration) and their order values are increased by a large amount, as a higher value would yield a greater abundance. As we do not have an inverse mapping from this new value to an amino acid, we find the substitute by taking “inspiration” from guide sequences, chosen as the top 10 highest abundance sequences. The residue with closest ordering value to the newly increased value (“O” in the example) is taken and this amino acid replaces the original one in the wildtype sequence. (c) The UMAP projection is a good approximation of the embedded manifold, as it generally correlates well with abundance (Spearman *p*‐value <1e‐308) (the plot is colored by density). Each point corresponds to the centroid of a sequence point cloud, projected through the learned UMAP function. The horizontal axis is normalized to the smallest and largest values in the set of projected points. The centroid of the lowest abundance sequence is marked with a black square and that of the highest abundance sequence with a black triangle. The approximation is worse for lower abundance sequences, as the red square should have appeared as the minimum ordering value. (d) Predicted abundance increase on sequences mutated with MGEM (black bars showing averages, with 95% confidence intervals). An increasingly higher number of residues with lowest ordering (2, 5, 10, 20 residues, as well as 10%, 20%, and 30% of the sequence) were selected in each scheme shown in the figure. The highest overall increase occurred for the scheme consisting of mutating the 20% lowest‐order residues. All schemes showed significantly higher values than random control (blue), which on average decreases predicted abundance. (e) The most important part of the sequence for the model is the N‐terminus, as measured by the embedded ordering value, here normalized to the inverse ranking of residue values (as the relative order is the important information) divided by sequence length. The plot shows the average such profile for sequences of length 200–400, the profiles of which were upsampled by linear interpolation to maximum length. (f) The high importance of the N‐terminus for abundance leads to fewer residues being mutated by MGEM, as a consequence of the embedded ordering values (shown in F). Except for the first few positions in the sequence, most amino acids in the leading 20% of the sequence are generally untouched (the leading M is avoided by MGEM). The plot shows for each amino acid the normalized MGEM substitution rate over sequence length bins spanning the leading 30% of sequences (computed over all sequences and mutation schemes). The position has been normalized to sequence length and binned to 2 decimals (resulting in 100 bins). For each amino acid, the number of times MGEM has replaced it in a bin was divided by the wildtype count of that amino acid in the same bin. The *z*‐scores of these values were obtained separately for each amino acid. (g) Average fraction of wildtype (left) and MGEM mutant (right) amino acid over the leading 30% of all mutated sequences (error bars showing 95% confidence intervals). The amino acids are colored by their normalized hydrophobicity (Cid et al. 1992), which highlights the overall mutation shift towards more hydrophobic proteins. The binning was performed as in F), that is, over 30 of the position 100 bins for each sequence.

Next, we hypothesized that our model could facilitate control over protein abundance by introducing targeted changes to the protein sequence. To achieve this, we developed a Mutation procedure Guided by an Embedded Manifold (MGEM), which enables us to navigate the Transformer model's embedded sequence manifold and perform individual amino acid substitutions that increase predicted abundance using only positional values derived from the embedded space. The approach involves traversing a uni‐dimensional UMAP projection of the Transformer encoder's high‐dimensional embedded space, which assigns a scalar importance value to each residue in a sequence based on its impact on protein abundance (i.e., as determined by both position and amino acid that the model learned) (Figure 2a). This is intended as a way to peer inside the neural network's black box and explore sequence space, allowing per‐residue comparisons of sequences (and their variations) in terms of predicted value. MGEM uses the projections and substitutes low‐importance residues in a starting wild‐type sequence with high‐importance residues from a set of guide sequences selected based on their topmost abundance levels (Figure 2b; see details in sections 4.10 and 4.11). Thus, borrowing important amino acids (as measured by their order in the UMAP projection) from highly abundant proteins makes the modified sequence “move” towards higher predicted abundance. This principle is based on the posited property of the high‐dimensional Transformer embedded space by which the sequence representations are approximately ordered (or “ranked”) according to the target value (Figure 2a). The per‐residue importance values obtained with UMAP are a good approximation of this ordering (Spearman's *ρ* = 0.8, *p*‐value <1e‐16) (Figure 2c), enabling the sorting of all residues on a univariate scale that spans all sequences, according to their importance towards prediction (see section 4.10). Our novel method relies on the learned relationship between sequences and changes wildtypes by deterministically substituting the individual amino acids deemed most impactful to abundance without relying on probabilistic or stochastic optimisation searches.

We next performed a series of in silico MGEM sequence perturbation experiments by introducing substitutions that would increase predicted protein abundance. This was done across the entire set of protein sequences in different substitution schemes, each consisting of changing a given number of lowest importance residues per sequence (a fixed number of 2, 5, 10, and 20 residues, as well as 10%, 20%, and 30% of residues in each sequence). We observed that MGEM enables control of target values (protein abundance) significantly more than a random control (paired *t*‐test, adj. *p*‐value <1e‐16 for all schemes) in which a random set of residues of the same size as the MGEM set for the given scheme was selected and mutated to random amino acids (Figure 2d). Indeed, on average, random mutations yielded a decrease in predicted protein abundance. The greatest MGEM increase was obtained when mutating 20% of the sequence, achieving an average 675% predicted abundance increase.

By inspecting MGEM mutants, we discovered that in terms of sequence position, the N‐terminus is the most important for abundance prediction. The average wild‐type embedded ordering (importance) profile peaks over the leading 20% of the sequence (Figure 2e), and as a consequence of the MGEM selection process, results in most amino acids being left unchanged in this region (Figure 2f). Additionally, there is a much shorter hotspot of frequently mutated amino acids at the very last positions of the C‐terminus. In accordance with other studies (Orth et al. 2010; Tuller and Zur 2015), this would suggest that the N‐terminus is generally evolutionarily optimized for expression efficiency, though our results point towards this optimisation having taken place at the amino acid level as well, besides the codon usage level and in terms of mRNA folding strength, which is in accordance with previous assessments (Tuller and Zur 2015). Indeed, the composition of the first 30% of sequences significantly differs from the composition of the full sequences (one‐sided hypergeometric test, *p*‐value <1e‐3), with the leading region enriched in Ala (A), His (H), Met (M), Pro (P), Gln (Q), Arg (R), Ser (S), Thr (T) (Table S5). The observation that distributions of substituted amino acids differ from the above (some are replaced uniformly across the entire sequence length) indicates the role of the amino acid's position and nature. In terms of replacement amino acids, we observed that the vast majority are A, G, and V (Figure 2g). In terms of physicochemical AAindex variables, mutants show significant perturbations (paired *t*‐test, *p*‐value <1e‐80) (see Table S6 and Figure S9), especially in indices that describe *polarity* (specifically amphiphilicity, with a 19% average decrease), *backbone conformation propensity* (with the largest index average decrease by 18% and the highest average index increase by 9%), and in the *preference for position at α‐helix cap* (average decrease by 5%), which suggests a change in the likely secondary structure and a shift towards higher hydrophobicity in the mutants.

### 2.4 Mutant proteins with high predicted abundance show greater stability at a lower metabolic cost

**FIGURE 3:**
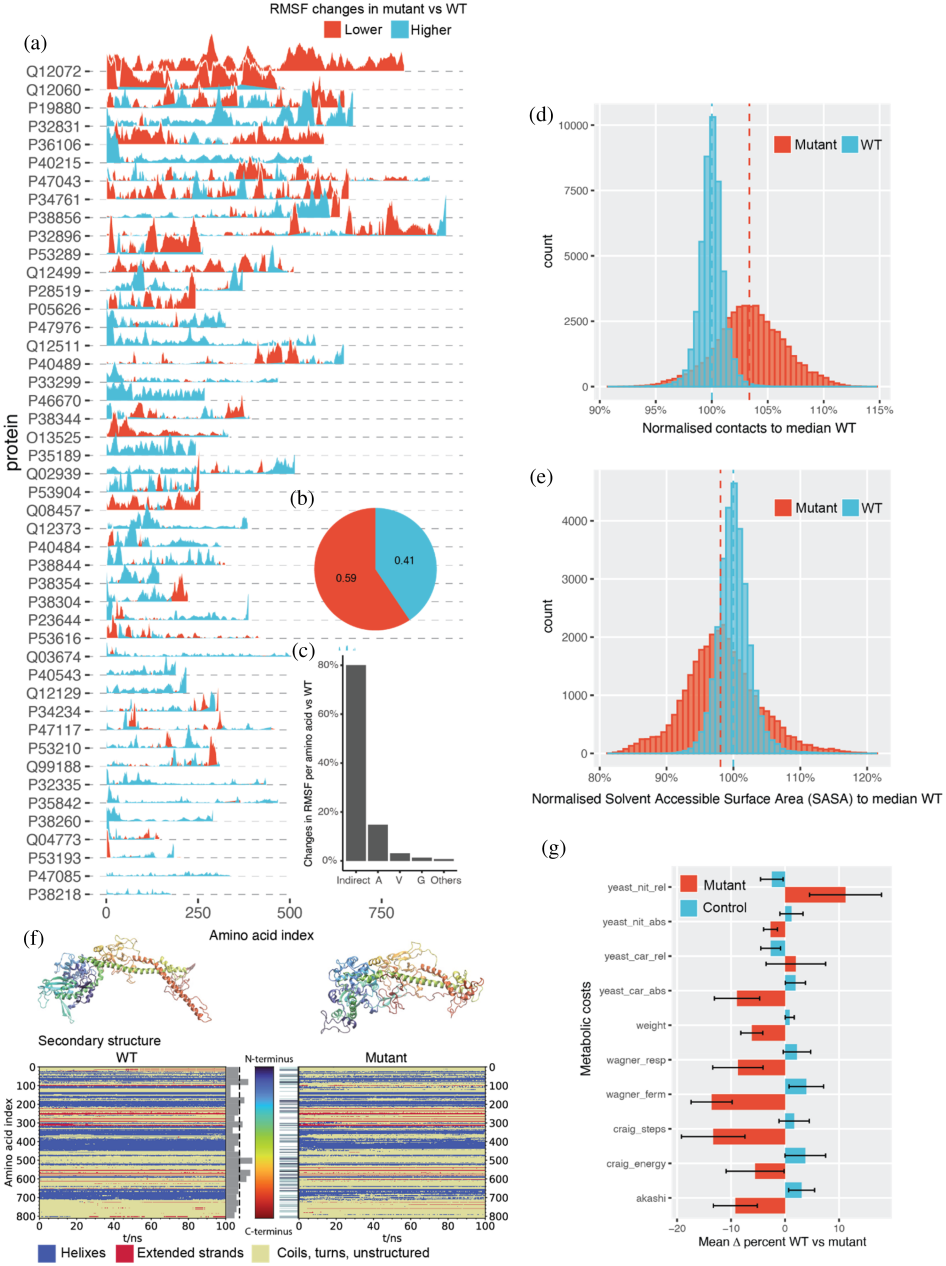
Abundant proteins exhibit higher conformational stability and are synthesized at a lower cost. (a) Differences between root mean square fluctuations (RMSF) between abundance‐increasing mutants and wildtype (WT) structures over 100 ns of molecular dynamics trajectory. (b) Fraction of atomic fluctuation that are at least 2 standard deviations lower in mutant (red) versus wt (blue). (c) Fraction of total significant (absolute *z*‐score >2) changes in RMSF per introduced mutation. Indirect denotes the regions of protein sequence with no mutations. (d) Comparison of contacts between WT and abundance‐increasing mutants. Normalization is done with reference to WT using frames after half of the 100 ns trajectory, contacts are considered at 8 Å proximity of the carbon backbone (section 4.14). (e) Comparison of solvent accessible solvent area (SASA) between WT and abundance‐increasing mutants. Normalization is done with reference to WT using frames after half of the 100 ns trajectory. (f) Structure (top) and DSSP plot (bottom) of the wildtype (left) and the mutant (right) of IOC2 yeast protein. The structures represent the last frame of the respective simulation (100 ns). The coloring denotes the amino acid index as shown by the color bar in the center (N‐terminus: blue to C‐terminus: red). In the DSSP plot, helical structures are highlighted in blue, extended structures in red and everything else (e.g., coil, turn, unstructured) in yellow. The bar plot represents the mutation rate per ~32 amino acids per bar; the dashed line represents the average mutation rate per bar. On the right‐hand side, the mutated spots are highlighted. (g) MGEM reduces protein cost. The average sequence costs of mutants obtained with MGEM (20% mutated sequence) show a significant overall decrease compared with random control (paired *t*‐test, *p*‐value <1e‐308), particularly in terms of synthesis costs (see also Table S7). The exceptions were two systemic costs from Barton et al. (2010), one having the lowest correlation with attention (12% cost increase on average), and the other having both weakly positively and negatively correlated subpopulations (2% cost increase on average).

The analysis of MGEM mutants indicates that sequences with increased predicted protein abundance were primarily obtained using non‐polar A, G, V amino acid substitutions (Figure 2g). We note here that substitutions are based purely on how the model ranks amino acids and their positions contributing to the abundance prediction within a given sequence. Alanine is known to stabilize helices, while glycine varies in its effects (Pace et al. 1998). Glycine can enhance rigidity in β‐turns (Trevino et al. 2007). Valine is common in thermophilic proteins (Panja et al. 2020), and alanine and valine substitutions often show similar helix impacts (Gregoret and Sauer 1998). Cysteine, infrequently substituted by our procedure (Figure 2g), is vital for thermostability due to its potential for disulfide bridge formation (Sevier and Kaiser 2002). Likewise, it has been observed that highly expressed proteins are often more thermostable (Luzuriaga‐Neira et al. 2023; Serohijos et al. 2012). Note that we use the term “stability” in referring only to thermostability, as the correspondence between thermostability and thermodynamic stability is not linear. Using our method, which allows for mutations that increase predicted protein abundance, we sought to determine if the model‐learned sequence‐to‐abundance mapping is anyhow linked to protein stability. To corroborate this, we applied molecular dynamics (MD) simulations to 100 pairs (mutant and wildtypes, WTs) of non‐membrane yeast proteins (Figure 2d, 20% mutation regime). Both mutated and their original WT versions were modeled using AlphaFold2 structures (section 4.12), and molecular systems were simulated for 100 ns. Our model does not account for 100% of protein (Figure 1c) abundance variation nor is aware of protein language, as such, there is a risk that introduced mutations could destabilize proteins (Johnson et al. 2024). Therefore, we only considered WT and mutant pairs that converged over 100 ns of the simulation trajectory (Figure S10 and section 4.12), resulting in ~46% of the simulations in our subsequent analyses. To quantify the degree of protein backbone conformational changes, we first started by comparing atomic position fluctuations, expressed as the standard deviation of residue alpha carbons across the entire course of the MD trajectory (root‐mean‐square fluctuations, RMSF) between mutant and WT sequences. Thirty‐three percent of converged systems showed significantly lower RMSF in comparison to WT proteins (Wilcoxon rank sum test, adj. *p*‐value <1e‐2) (Figures 3a and S11). Decreases in protein backbone fluctuations are a sign of protein rigidity (Karshikoff et al. 2015), which is frequently pronounced in thermophiles when compared to their homologous mesophilic variants (Frappier and Najmanovich 2015; Rader 2009; Radestock and Gohlke 2008; Sen and Sarkar 2022; Zhang and Lazim 2017); reducing protein's flexibility is often used strategy for increasing protein thermostability and half‐life (Pucci et al. 2014; Rader 2009; Radestock and Gohlke 2008; Yu and Huang 2014; Zhang and Lazim 2017). Fifty‐nine percent of atomic fluctuations of mutants predicted to be highly abundant were at least two standard deviations lower than the corresponding positions of the WT trajectory (Figure 3b). About 81% of mutations had no direct impact on atomic fluctuations, that is, we observed changes in fluctuations in residues as high as two standard deviations away from corresponding WT positions with no mutations, suggesting that changes in atomic fluctuations caused by abundance‐changing mutations affect overall global protein dynamics, rather than just local residues (Figure 3c).

Although large structural changes from mutations can destabilize proteins (Luo and Baldwin 2001; Zhang and Lazim 2017), backbone conformational changes do not directly indicate protein stability. To corroborate further, we inferred the effects of predicted abundance‐increasing mutations using the DeepET model (Li et al. 2022) trained on organism growth temperature and protein melting data (Jarzab et al. 2020; Leuenberger et al. 2017). We estimated T_OGT_ (OGT, organism growth temperature, which is highly correlated with protein melting temperature Tm (Li et al. 2022)) in mutants. Increasing protein abundance showed a significant (paired *t*‐test, *p*‐value = 0.021) average 17% increase in T_OGT_ compared to WT sequences (section 4.13 and Figure S12). We also examined intermolecular interactions, specifically the number of contacts between neighboring amino acids (section 4.14). Stable proteins with robust hydrophobic cores generally have more native contacts (Dill et al. 2008). In our comparison, 84% of the mutants predicted to be highly abundant exhibited significantly more contacts than corresponding wildtypes (Wilcoxon rank sum test, adj. *p*‐value <1e‐4) (Figures 3d and S13). It is known that proteins which easily denature expose their hydrophobic core, resulting in lost hydrophobic interactions and increased solvent accessibility (Eisenhaber et al. 1995; Pace et al. 1996; Zhang and Lazim 2017). Investigating the effects of A, G, and V substitutions on hydrophobic cores, we computed the Solvent Accessible Surface Area (SASA) for all proteins. We found a significant decrease (Wilcoxon rank sum test, *p*‐value <1e‐4) in SASA for abundance‐increasing mutants versus wildtypes, corroborating the link between rigidity, conformations that are also observed in thermotolerant mutants (Frappier and Najmanovich 2015; Rader 2009; Radestock and Gohlke 2008; Sen and Sarkar 2022; Zhang and Lazim 2017), and abundance (Figure 3e).

Next, we closely examined the strongest effects of mutations as observed in the ICO2 protein (UniprotID: Q12072), which had the highest RMSF perturbations (Figure 3a). Although the mutant and WT IOC2 started similarly, they diverged dynamically over 100 ns of simulation (Figures 3f and S14). The stable core, largely less mutated, differed from the more mutated C‐terminal region (Figure 3f, bar plot). A notable change was the breaking of an alpha‐helix in the mutant, enabling the C‐terminus to fold closer to the protein core. This change led to an increase (WT: 53.0%, mutant: 59.9%; Mann–Whitney *U* test, *p*‐value <1e‐16) in the median unstructured secondary structure (Figure 3f, DSSP) but formed a more compact shape than its WT counterpart. Despite imperfect alignment in the C‐terminal region, an overall increase in hydrophobicity is seen in the mutant (mean −0.07 with the WT vs. 0.17 with the mutant, Mann–Whitney *U* test *p*‐value <1e‐4), reflected in a reduced RMSF (Figures 3a and S11).

Finally, we analyzed the metabolic cost implications of predicted abundance‐increasing mutants compared to wildtypes, given concerns that increased protein copies might affect fitness (Agozzino and Dill 2018). Overall, predicted abundance‐increasing mutant metabolic costs decreased significantly compared to random controls (Figure 3g, paired *t*‐test, *p*‐value <1e‐16). The most notable reductions were in synthesis under fermentative growth (*wagner_ferm*, −14% average) (Wagner 2005) and biosynthetic steps from central metabolism to the resulting amino acid (*craig_steps*, −13% average) (Craig and Weber 1998). Both factors had a strong inverse relationship with Transformer attention (Figure 1e and Table S1), confirming that the embedded space ordering (Figure 2a) and the model's attention indirectly pick up the same evolutionary phenomenon. The exceptions were the impact of the relative change of the amino acid requirement on the minimal intake of ammonium (Barton et al. 2010) (*yeast_nit_rel*, 11% increase on average), which had the lowest correlation with attention, and the impact of the relative change of the amino acid requirement on the minimal intake of glucose (Barton et al. 2010) (*yeast_car_rel*, 2% increase on average, see Table S7 for a full list). In summary, the significant cost reduction observed is especially striking since neither the neural network nor the MGEM procedure were specifically trained with cost as a factor. This suggests that the neural network inherently recognized the connection between sequence cost and protein abundance, aligning with earlier observations on the cost‐effective metabolism of highly abundant proteomes (Akashi and Gojobori 2002).

## 3 DISCUSSION

Intracellular protein levels are determined by a delicate interplay of synthesis, regulation, and degradation. Despite the vast codon variability and regulatory sequence divergence seen both within and between species at the DNA level (Cutter et al. 2006; Plotkin and Kudla 2011), the conservation of protein ortholog abundances across diverse evolutionary lineages suggests an evolutionary imprint on amino acid sequences (Laurent et al. 2010; Schrimpf et al. 2009; Tuller et al. 2010a). While intricate cellular dynamics play a role in immediate protein concentrations, significant evolutionary information likely resides within the primary sequence itself. Supporting this notion, our analysis of over 800 proteome datasets (Huang et al. 2023), representing species from the entire tree of life shows that in at least 38 species, including mouse and human protein, the amino acid sequence predicts over half of protein abundance (Figure 1a,b).

Given that proteins have such a changing nature it is natural to ask how it is possible to predict the dynamic nature of protein abundance from a constant protein sequence? To explain this phenomenon we analyzed cross‐experimental copy number variation using a consolidated proteomics dataset from a comprehensive list of yeast studies (Ho et al. 2018). It appeared that the gene‐wise dynamic range of protein abundances spanned an average of 5 orders of magnitude, while individual protein expression values for 95% of measured yeast proteins vary only within one relative standard deviation (RSD) across all experimental conditions (Figure S6). While proteins vary across experimental conditions, their copy numbers on average stay within the same order of expression values, explaining the deterministic nature of proteomes. A similar phenomenon has been observed previously with mRNA levels encoded in the DNA sequences (Agarwal and Shendure 2020; Zrimec et al. 2020). These results led us to postulate that amino acid sequences may inherently encode protein abundance.

By observing that amino acid composition across deciles of the dynamic range of protein expression is rather uniform (Figure S6), we inferred that the amino acid arrangement in the sequence and not merely composition coding for protein abundance (Figure S5). To study this further, we trained a deep neural network from scratch to predict protein abundance accounting for over half of the variability in abundance of the entire proteome dynamic range (Figure 1c, *R* ^2^ test = 56%). Additional validation that it is a sequence that encodes for abundance came from our model failing to predict shuffled sequences (Figure S5) and attention profiles from these randomized sequences no longer correlated to protein features (Figure 1e,f).

It is naturally intriguing to make more explicit how positional and compositional features differ towards the prediction of protein abundance. Here, one is frustrated by the significant overlap in “information” and the remaining unknowns regarding the physical dependencies between all relevant variables, which are likely to be complex and nonlinear (as is the case between amino acid composition and codon usage bias for instance, as selection at the amino acid level influences codon usage (Błażej et al. 2017; Morton 2001)). To try to elucidate what our model has learned in terms of structural and compositional information at the nucleotide level (as the question arises of how much of this carries over to its predictions), we examined the associations with mRNA folding strength (Kertesz et al. 2010) and tRNA adaptation index (Tuller et al. 2010b), as both are known to strongly correlate with protein abundance. To better understand what each variable contributes independently, we calculated partial correlations to the model predictions and residuals separately (section 4.15). We saw that the information captured is split between the predictions and residuals, codon usage being the factor that (independently) contributes to both (Figure S15). For predictions especially, mRNA folding strength does not contribute significantly. Codon usage (as tRNA adaptation index) thus explains only about 45% of our model results.

The contributions of the various protein features on abundance have been studied mostly in isolation using linear models based on numerical summarization of nucleotide or amino acid composition, giving predictors of varying strengths, of which the most significant for *S. cerevisiae* are mRNA levels (*R* ^2^ = 52% on average), codon usage bias (*R* ^2^ = 56%) translation rates (*R* ^2^ = 58% on average) (Cascarina and Ross 2018; Ho et al. 2018; Riba et al. 2019; Vogel and Marcotte 2012; Zur and Tuller 2012; Zur and Tuller 2013). However, given the dynamic nature of protein synthesis, degradation processes, and their interactions, nonlinear models that integrate or abstract over the multiple levels are desired, especially given the loose coupling between some of these (e.g., the dynamic range of protein abundance is larger than that of mRNA and the former have longer half‐lives (Vogel and Marcotte 2012)). Thus, to decipher the biological insights gained by the neural network in predicting protein abundance, we analyzed the patterns within the Transformer self‐attention mechanism. Notably, attention profiles showed correlations with known protein abundance determinants (Figure 1e), including amino acid synthesis costs, suggesting that the model recognized the cell's energetic currency concerning protein synthesis. The attention mechanism identified multiple associations between residues throughout the sequence, hinting at the neural network's ability to discern overarching structural and physicochemical sequence patterns (Figure 1f). Our analysis further revealed that the network prioritizes regions with distinct secondary structure elements and functional domains when predicting protein abundance (Figure 1g,h). Moreover, the correlations found between attention, sequence, and physicochemical properties like polarity and hydrophobicity underscore the potential relationship between protein abundance and a protein's structural features (Figure 1f). These findings, together with the validation using randomized sequences, lend more credence to the network having learned sequence patterns and interactions, complementing the various other predictors based on compositional summarization.

While attention links specific residue positions to abundance prediction, understanding the encoder embedded space—a reflection of the sequence grammar grasped by the Transformer—is more challenging. This high‐dimensional space encapsulates intricate sequence semantics but is not straightforward to interpret, resulting in a “semantic gap” between features and (human) meaning, often seen in deep neural networks (Duan and Kuo 2021; Wiegreffe and Pinter 2019). Thus, to enhance our model's explainability, we introduced the MGEM analytical framework. It simplifies the sequence space exploration by first establishing a one‐dimensional reference (Figure 2a,b), then guiding mutations towards target sequence regions. Unlike methods that can produce unreliable predictions (predictor pathologies) (Linder et al. 2020; Nguyen et al. 2014; Szegedy et al. 2013) or local minima problems (Bogard et al. 2019), MGEM deterministically modifies sequences based on their mapped target value, offering a deterministic solution for amino acid substitutions beneficial for multiple applications.

We applied the MGEM framework to perform a series of control‐perturbation experiments to identify amino acids and protein properties that are intrinsically related to abundance (Figure 2a,b). Compared to the random control, which resulted in a decrease in protein abundance, MGEM‐guided mutations achieved an average abundance prediction increase of over six times compared to the wild‐type sequences (Figure 2d). By inspecting MGEM mutants, we discovered that in terms of sequence position, the N‐terminus was the most important, with the majority of amino acids remaining unchanged in this region (Figure 2e,f). This suggested that the N‐terminus is generally evolutionarily optimized for expression efficiency, which is known to impact translation efficiency (Verma et al. 2019), and which also supports why it is widely used for protein expression optimization (Wang et al. 2022; Wu et al. 2020; Xu et al. 2021). A short hotspot at the very last position in the C‐terminus was frequently mutated, which is known as a signal involved in protein degradation (Correa Marrero and Barrio‐Hernandez 2021; Weber et al. 2020). Besides the C‐terminus, however, most of the amino acids were substituted uniformly across the entire sequence length, based solely on their model‐induced importance ranking, mainly with the amino acids A (alanine), G (glycine), and V (valine) (Figure 2g), which are hydrophobic. The introduction of hydrophobic amino acid residues into protein secondary structural components, such as helices, sheets, and turns, is known to affect a protein's thermostability (Gregoret and Sauer 1998; Pace et al. 1998; Panja et al. 2020). We therefore wanted to see if our model captured a link between the predicted increase in abundance and protein structure, and hence its stability.

We investigated this using extensive molecular dynamics (MD) simulations, an established technique for studying protein dynamics at the atomic level (Pikkemaat et al. 2002; Zhang and Lazim 2017). Our data, derived from 200 MD simulations of randomly chosen yeast proteins, showed that the majority of abundance‐increasing mutations had increased the number of protein contacts and reduced solvent accessibility as reflected in reduced root mean square fluctuations (Figure 3a,d,e), phenotypes representative of thermostable proteins (Kumar et al. 2000; Razvi and Scholtz 2006; Robinson‐Rechavi and Godzik 2005) (Figures 3d,e and S13). We independently confirmed using a neural network trained on measures of thermostability (Li et al. 2022) that abundance‐increasing mutations increase predicted protein stability temperatures (Figure S12). In addition, we performed a proteomics experiment on the most pronounced protein (ICO2 protein, UniprotID: Q12072) identified from MD experiments, by comparing protein expression fold‐changes in mutant and wildtype between growth phases (Data S1 and section 4.16). The results indicate that the mutant has up to 50% lower protein degradation propensity in comparison to the wildtype, which could be due to increased stability. The abundance increase observed here is comparable to the effects due to open reading frame (amino‐acid synonymous) nucleotide substitutions performed on non‐native proteins in a *S. cerevisiae* host. Using different techniques and changing varying fractions of their target gene coding sequences, differences of on average 3‐fold in protein expression have been achieved with such nucleotide substitutions (Ben‐Yehezkel et al. 2015; Cripwell et al. 2019; Kim et al. 2013; Lanza et al. 2014). We note that the aim of the current work was to investigate a fundamental relationship between sequence and abundance rather than use amino acid mutation strategy as a way to engineer protein expression (van den Berg et al. 2012; van den Berg et al. 2014). While we kept codon frequencies the same as in the wildtype strain, focusing solely on amino acid substitutions without modifying native gene regulatory regions, for example, promoters, likely leaving gene synthesis, transcription, and translation unaffected, however observations from a single experiment should be approached with caution, that is, it would require much more experimentation to figure out if the introduced mutations directly reduce in vivo protein degradation via stabilization of its conformation or operate through other mechanisms. Nevertheless, these results together with the predictions from MGEM sequence perturbation experiments, as well as the results from MD simulations align well with previous observations that highly abundant proteins are generally more thermostable (Agozzino and Dill 2018; Serohijos et al. 2012; Serohijos et al. 2013; Yang et al. 2010). This phenomenon is often explained by the so‐called misfolding avoidance hypothesis and related hypotheses, which have dominated evolutionary discussions for the past decade, all aimed at explaining the slower evolutionary rates observed with highly abundant proteomes (Pál et al. 2006; Zhang and Yang 2015). An alternative explanation for the slow evolution of abundant proteins suggests that higher benefits come with higher costs (Cherry 2010; Gout et al. 2010; Zhang and Yang 2015). However, our findings indicate that proteins with mutations enhancing their rigidity, and potentially stability (Figure S12), are not only more abundant but also more cost‐effective to produce. This would explain their evolutionary advantage, as a structurally stable protein incurs fewer synthesis‐associated costs to maintain consistent protein levels. Finally, relating back to the model expressing protein abundance (Figure S1) as the joint contribution of translation efficiency and protein half‐life, we see our Transformer model, in conjunction with the MGEM procedure, recovers synthesis cost (from sequence), rigidity (from molecular dynamics) and thermostability (DeepET model) as a link to abundance.

In conclusion, while the primary goal of our study was to investigate the relationship between a protein's amino acid sequence and its abundance by interpreting learned latent features of a neural network, our analysis revealed connections between amino acid sequence, protein abundance, and metabolic cost related to protein thermostability and synthesis. Remarkably, even without explicit conditioning on synthesis cost, both our Transformer model and MGEM procedure succeeded in uncovering these latent relationships. This demonstrates the power of deep neural networks to decode complex biological systems. By manipulating the deep model's semantics of these latent relationships, we unintentionally produced sequences optimized for cost. We demonstrated in silico that mutations leading to increased predicted abundance also have evolutionary advantage through reducing the metabolic costs of protein synthesis and at the same time making proteins more rigid. In addition, the MGEM approach opens new avenues in protein engineering by providing a robust, targeted method for amino acid substitution mapped to any continuous (real‐valued) property. This has the potential for the design of proteins that are not only functionally efficient but also metabolically cost‐effective, beneficial for biotechnological applications as well as for facilitating interpretation of disease related mutations (Beltran et al. 2024; Topolska et al. 2024). While no single theory can likely fully explain the complex relationships between protein sequence, abundance, synthesis and stability, our work identifies a critical link among these factors. By integrating insights from neural network predictions, extensive MD simulations, we propose a hypothesis that suggests the evolutionary advantage of stable, abundant proteins: they may offer functional efficacy at a reduced synthesis cost.

## 4 METHODS

### 4.1 Random forest abundance model as synthesis and degradation

Ribosome profiling data—specifically, ribosome density *R* (the number of ribosome‐protected fragments as RPKM) and mRNA abundance *m* (as RPKM)—from Weinberg et al. (2016) and protein half‐life values from Christiano et al. (2014) were used to predict the median protein abundance values from the Ho et al. dataset (Ho et al. 2018). Intersecting these datasets and filtering out effectively zero (lower or equal to float32 machine epsilon 1.192e‐07) and missing values resulted in a set of 3574 protein values. All variables were log10‐transformed. Translation efficiency *TE* (Weinberg et al. 2016) was calculated as *R*/*m* (indeed, log10(*R*) − log10(*m*)). A random forest regression model using the scikit‐learn (Pedregosa et al. 2011) implementation was trained on *TE* and half‐life to predict protein abundance, using 20% of the data (715 proteins) as a hold‐out test subset. The best random forest parameters, found through a grid search on the training subset using 5‐fold cross‐validation, were *n_estimators* = 200, *min_samples_leaf* = 50, and *max_features* = 1.

### 4.2 Training the ESM embeddings model on PaxDb

Protein sequences and abundance measurements were downloaded from PaxDb on August 7th, 2024. Organism specific data sets were constructed by combining experimental abundance values and computing medians for each gene. For *H. sapiens* the experiments were also split into tissue specific and cell line specific experiments, for which medians were calculated, resulting in 57 and 42 data sets, respectively. After this process, organisms with less than 300 experimental values were dropped. For computational simplicity, sequences longer than 2048 amino acids were also removed. To remove data leakage that could potentially arise due to sequence homology, MMseqs2 was used to cluster the sequences at 30%. For each respective data set, 20% of the clusters containing single sequences were then set aside as test sequences for the respective organism and the remaining 80% were used for training. ESM‐2 was used to calculate average embeddings for all sequences (Lin et al. 2023) in each organism specific data set. To make the abundance value mass‐centered before training, Box‐Cox transformations were used with lambda values calculated based on the expectation maximization procedure on the training set partition of the data. The neural network models use two hidden layers with 512 and 128 neurons, respectively, and ReLU activation. A dropout of 0.3 was applied after the first hidden layer. The output used a single neuron with linear activation. The models were trained using the Adam optimizer with a learning rate of 0.001, beta1 = 0.9, beta2 = 0.999, and mean squared error (MSE) as the loss function. The performance of the models was evaluated by calculating the coefficient of determination (*R* ^2^) between the predicted values and the Box‐Cox‐adjusted values.

### 4.3 Neural network training

*Saccharomyces cerevisiae* (strain S288C) protein sequences were obtained from the UniProt (UniProt Consortium 2019) reference proteome UP000002311 on 20th January 2020. To avoid technical challenges when training neural networks, we restricted the set of proteins to those with a length between 100 and 1000 residues (yielding 5202 out of 6049 proteins). The intersection of this set with the proteins with available abundance values from Ho et al. (2018) resulted in 4750 unique sequences in our initial sequence‐abundance dataset. To assemble the final dataset we added repeated measurements for each protein sequence, namely each sequence appeared up to 21 times, each time with a different experimental target value from the Ho et al. dataset, as in a regression with replicates, resulting in 99,603 training examples used as input/independent variable. In order to steer the model towards learning sequence (positional) information, as opposed to amino acid composition, subsequently, for each sequence, a shuffled version was introduced with an “effective null” target value, a very small fractional value of 1e‐5 (the unit for absolute abundance is molecules per cell), to allow for power transformations, resulting finally in 199,206 sequences (thus, up to 21 shuffled versions of each unique sequence appear as counter‐examples). This was performed in order to expose the neural network to nonsense counter‐example sequences so that it may learn to distinguish and to facilitate sequence interpretation, similar to training for classification problems (Elliott et al. 2021; Gulshad and Smeulders 2020) (here, with real and nonsense classes) or similar to using decoy sequences for distinguishing signal from noise in mass spectrometry (Käll et al. 2008). The data was randomly partitioned as 80% training, 10% validation, and 10% test, by splitting on unique sequences, that is, ensuring repeated measurements of the same sequence were placed in the same data partition to avoid data leakage. Protein sequences (X's/independent variable) and their corresponding target raw abundances (Y's/dependent variable) were loaded as‐is to model as input lists without masking. To make the abundance distribution mass‐centered, the preprocessing was configured to Box‐Cox transform the raw abundances with *λ* = −0.05155 using the expectation–maximization procedure as implemented in SciPy, on data based on medians of the initial dataset.

The training task's preprocessing routine tokenized the sequences with the TAPE IUPAC (Rao et al. 2019) tokenizer, each amino acid being assigned a unique integer value and the sequence flanked with special start and stop integer tokens. The TAPE (Rao et al. 2019) implementation of the BERT *ProteinBertForValuePrediction* class was adapted for the model training. The model was trained as a regression task to minimize mean squared error (MSE). The model performance reported here was calculated by taking the median abundance across experiments for the proteins in the hold‐out test set (436 values), as the test set obtained as above contained sequence repeats. The coefficient of determination was calculated on median values of the hold‐out test using the Scikit‐learn function. Hyperparameters search was performed using the BOHB algorithm (Falkner et al. 2018) of the HyperBand scheduler (Li et al. 2017) provided by the Ray library (Moritz et al. 2018). Details about model architecture and hyperparameters are provided in Tables S9 and S10. The best hypermodel thus found was then retrained. The best model consisted of 8 attention layers with 4 heads each (see Table S8). The model was trained and optimized on a multi‐GPU cluster using a mixture of A100 and V100 NVIDIA GPUs.

### 4.4 Attention profile analysis

As it is generally unclear (Rogers et al. 2020) at which depth one might find lower or higher level features in such architectures, we considered all non‐redundant attention profiles for a given sequence when measuring matches. Specifically, as Transformers are known to have relatively high redundancy (i.e., different layers and attention heads learn very similar weights), we performed pairwise Pearson correlation of attention matrices from all layers and heads and kept only those that were uncorrelated (*r* < 0.01) with the majority (at least 90%) of other matrices, for each sequence. This left on average 4 non‐redundant attention matrices per sequence. Moreover, attention matrices exhibited strong asymmetry (see Figure S3), often consisting of effectively uniform vertical streaks (i.e., the majority of residues “attend to” a single residue near‐uniformly), thus making the “attended‐by” values more informative (i.e., which residues receive such attention from all others). These “attended‐by” values were averaged to produce one‐dimensional attention profiles, which could be correlated with various per‐residue measures. To match against qualitative data such as protein domains, we extracted residue attention *patterns* by keeping only the sequence positions with an attention value z‐score of at least 1 in the corresponding profile to keep only those positions with the most signal.

### 4.5 Cost analysis

Per‐residue cost profiles were computed for all proteins in the dataset (*N* = 4750) using the *S. cerevisiae* amino acid costs from Barton et al. (2010), with the exception of *yeast_sul_abs* and *yeast_sul_rel*, which were deemed trivial for this task since they featured zero cost for all but a few amino acids. These profiles were then Pearson‐correlated to all attention profiles for each protein (on average 4 attention profiles per protein), keeping only the maximum correlation with *p*‐value <1e‐5 for each protein, since we do not know beforehand which head will give the strongest response for a given input sequence, as the attention information is distributed across all heads. The *p*‐value was set using the Bonferroni correction for multiple testing at a target threshold of 0.05, thus resulting in 0.05/4750 = 1.053e‐05. The same procedure was repeated with randomly shuffled versions of the same sequences to produce control distributions.

### 4.6 AAindex correlations

All 544 AAindex measures (https://www.genome.jp/aaindex/, release 9.12006) were computed on a subsample of 1000 *S. cerevisiae* proteins using the R package Bio3D 2.4‐3 (Grant et al. 2006). An average absolute correlation matrix was computed across the protein sequence subset and the AA indices were filtered using the R *findCorrelation* function (with a cutoff of 0.5) from the *caret* package 6.0–88, to only keep an non‐redundant subset of 18 AA indices: BUNA790103, FINA910104, GEOR030103, GEOR030104, LEVM760103, MITS020101, NADH010107, NAKH920107, PALJ810107, QIAN880138, RICJ880104, RICJ880117, ROBB760107, TANS770102, TANS770108, VASM830101, WERD780103, WOEC730101. These per‐sequence profiles for these indices were then computed for all proteins in the dataset (*N* = 4750) and Pearson‐correlated to all attention profiles. Only the maximum correlation with *p*‐value <1e‐5 was kept for each protein. The *p*‐value was set using the Bonferroni correction for multiple testing at a target threshold of 0.05, thus resulting in 0.05/4750 = 1.053e‐05. Note that the polar requirement (WOEC730101) was not part of the non‐redundant list and was added manually due to its frequent description in the literature and the low correlation (*r* < 0.4) to the other indices. The resulting correlation distributions were filtered to only those AA indices with an absolute mean correlation of above 0.3 across all proteins. The same procedure was repeated with randomly shuffled versions of the same sequences to produce control distributions, except the correlation threshold was removed in order to show the small resulting values. As a result, all 18 AAindex variables are plotted for the control.

### 4.7 Secondary structure analysis (DSSP)

Available *S. cerevisiae* PDB files (4745) generated by AlphaFold2 were downloaded from RCSB‐PDB (on 2022‐03‐18). For each of these, DSSP 3.0.0 annotations were obtained using the BioPython 1.79 (Cock et al. 2009) *dssp_dict_from_pdb_file function*. For each protein and all its attention profiles (4/protein, on average), DSSP annotations at positions with attention z‐scores >1 were counted. To avoid small numbers for significance testing, only structures with counts >10 were kept. For all attention profiles, one‐sided hypergeometric tests with a threshold *p*‐value of 0.05 were performed both for enrichment and depletion of structure annotation counts, against the total background count of annotations across all proteins. Finally, this was summarized as the number of proteins that have attention profiles enriched or depleted in each type of DSSP structural annotation.

### 4.8 Domain analysis

Each InterPro domain was overlapped with the attention patterns produced for its protein (i.e., the positions of the sequence with attention *z*‐score >1), recording the highest overlap fraction (i.e., the largest fraction of *attended‐to* domain residues) among all patterns produced for the sequence (output from all network layers and heads). To have a balanced control set, only domains that stretched to at most 50% of their protein length were kept (18,000 domains), so that the attention coverage inside the domain could be weighted against that outside of it. This was done (for each domain) by taking the number of high‐attention positions outside the domain and dividing it by the number of times the domain could fit in the outside region (i.e., the number of windows the same length as the domain). This yielded an expected count corresponding to repeatedly randomly sampling subsequences the same length as the domain. The coverage fractions were taken as the number of high‐attention positions (either in the domain or the expected value outside) divided by the length of the domain. To assess the significance of the difference in domain coverage fraction distribution between attention and control, we performed a two‐sided Wilcoxon signed‐rank test separately for each domain member database. The adjusted *p*‐values were <0.05 for 10 out of 12 member databases, where SFLD and HAMAP differences were not significant.

### 4.9 GO term enrichment analysis

The GO enrichment analysis for domains that overlap with attention was performed considering the proteins that have well‐covered domains (>=30% of their positions overlapping attention patterns) against the full set of proteins, with the Python library GOATOOLS 1.0.15 (Klopfenstein et al. 2018) using the Holm‐Bonferroni *p*‐value correction method and a significance threshold of 0.05. To summarize the results, GOATOOLS was used to obtain yeast GO slim terms (Table S4).

### 4.10 Embedded ordering

In order to assess how individual amino acids in a sequence affect the abundance prediction, we probed the embedded space that the Transformer encoder maps to. We call an *embedded ordering* the parametric UMAP projection (Sainburg et al. 2020) that we trained to map from this space down to a one‐dimensional scale. The encoder's embedded space contains 1024‐dimensional point clouds (one cloud for each sequence) (Figure 2a), with every amino acid being assigned a (1024‐dimensional) point. And because the Transformer uses a learned positional encoding, each residue in the sequence may be assigned a different value depending on position (i.e., regardless of the type of amino acid). From this space, a relatively simple feed‐forward network (2 weight‐normalized linear layers) is used for predicting values on the real line (Box‐Cox‐transformed protein abundances). The fundamental assumption of our construction is that (good) training induces a structure on the embedded encoder space that reflects the total order of abundance values (i.e., all scalar values are comparable and arranged in a strict succession). Under this assumption, we posit there exists a relatively low‐dimensional manifold on which a geodesic connects all points in the (full) embedded space, resulting in an arrangement from lowest‐prediction‐value point clouds to highest‐prediction‐value point clouds (Figure 2a). The geodesic thus gives a total order within the embedded space. To retrieve a manageable approximation of the geodesic (and thus, of the order), we trained a parametric UMAP projection down to one‐dimensional space. The embedded ordering thus constructed assigns a scalar value to each residue in the sequence, reflecting its contribution to the prediction. Moreover, these scalar values reflect a global ranking across the entire sequence space, that is, lower abundance sequences will have residues with overall low order values, and the converse for higher abundance sequences. This enables easy assessment of the importance of each residue and enables mutation procedures.

The training set for the parametric UMAP consisted of the embedded start token point of each sequence, as information from the entire sequence is “routed” through these network nodes in the attention layers, and 10% of these were kept as a hold‐out test set. The training was performed over multiple values of the UMAP number of neighbors hyperparameter, spanning an inclusive range from 1% to 25% of the number of sequences in the training set (aiming to balance local versus global structure). The performance was evaluated as the Spearman correlation between the centroids of the UMAP‐projected point clouds and the corresponding abundance targets over test sequences.

### 4.11 Mutation guided by an embedded manifold

The guided mutation was performed by sorting the residues according to their embedded ordering value and selecting the lowest of these for substitution, a different number for each scheme: the lowest 2, 5, 10, and 20 residues in each sequence, as well as the lowest 10%, 20%, and 30% of residues in each sequence. The 10 highest abundance sequences were selected as guides. This gives a pool of 4480 points distributed on the higher range of ordering values, available for substitution. For each residue selected to be substituted, its order value was increased by a large value, set as the width of the interval containing 99% of the embedded ordering (UMAP‐projected) values, intuitively inducing a large shift in contribution to the prediction. To obtain a substitute residue that would match this shifted value, the guide sequences were used. The residue with the closest ordering value to this shifted value in each guide sequence was then chosen as a substitution candidate. This substitution was repeated for 10 guide sequences, and the one resulting in the highest prediction increase was finally selected. Both, for the guided and the random substitution, the leading M residue was avoided. Random control was performed by choosing random residues (the same number as for each respective scheme) and substituting them with random amino acids.

### 4.12 Molecular dynamics simulations

We randomly subsampled 100 proteins with an increased abundance of at least 100% (from the 20% mutation regime; Figure 2d), ignoring transmembrane proteins. We applied molecular dynamics (MD) simulations to 100 mutated non‐membrane yeast proteins showing higher abundance (Figure 2d; 20% mutation regime). Structures were generated for mutated sequences and their corresponding wildtypes using AlphaFold2 (Jumper et al. 2021). The structures were generated utilizing the full big fantastic database (BFD) and all five CASP 14 models (Jumper et al. 2021). The structures with the highest average pLDDT score for each sequence were then selected for molecular dynamics simulations. Simulations were carried out using the GROMACS simulation package 2022 (Berendsen et al. 1995; Hess et al. 2008; Van Der Spoel et al. 2005), the AMBER99*‐ILDN force field (Aliev et al. 2014) and the TIP3P water model (Jorgensen et al. 1983). The protein was centered in a dodecahedron box with 1 nm distance to the box's boundaries, solvated and neutralized by adding ions. The energy of the solvated system was minimized using the steepest descent algorithm (steps = 50,000, emtol = 1000 kJ/mol/nm, emstep = 0.01). Afterwards, the system was equilibrated for 100 ps in an NVT ensemble, followed by a 100 ps equilibration in an NpT ensemble. For the productive run, an NpT ensemble was chosen using the Parrinello‐Rahman barostat (ref_p = 1 bar, tau_p = 2 fs, compressibility = 4.5e‐5 bar^(−1)^) (Parrinello and Rahman 1981). The temperature was set to 300 K using the v‐rescale thermostat (tau = 0.1) (Bussi et al. 2007). For all steps periodic boundary conditions were applied in all dimensions. For the simulations, a leap‐frog integrator (Hockney et al. 1974) with a time‐step of 2 fs was chosen. Covalent bonds involving hydrogens were constrained using the LINCS algorithm (lincs_iter = 1, lines_order = 4) (Hess et al. 1997). Short‐range non‐bonding interactions were cut off at 1 nm. For the van‐der‐Waals interactions, a Verlet‐cutoff scheme (ns_type = grid, nstlist = 10 steps, DispCorr = EnerPres), for the electrostatic interactions a Particle‐Mesh‐Ewald summation (pme_order = 4, fourierspacing = 0.16 nm) (Darden et al. 1993) was applied. For each protein, simulations were run for 100 ns. Protein coordinates were written to file every 1 ps. Simulations were considered converged if the RMSD was within a 10% error margin for 80% of the time points in the final quarter (Figure S10). Only these converged simulations (entire 100 ns) were selected for RMSF profile comparisons (Figure 3a).

### 4.13 Thermostability prediction based on T_OGT_

The optimal growth temperature and optimal enzyme activity temperature for the wildtype and abundance increasing mutant sequences were predicted using models developed by Li et al. (2022). For predictions, the model required sequences to be no longer than 512 residues, as such 21 proteins exceeding this length were excluded from the analysis. To assess the significance of the difference in predicted temperatures between the wildtype and variant sequences, a paired *t*‐test was conducted.

### 4.14 Analysis of MD simulations

For the analysis, first the periodic boundary conditions were fixed and afterwards, the frames were rotationally and translationally fitted onto the protein atoms of the last frame of the trajectory using a least‐square fit as implemented in GROMACS *gmx trjconv*. RMSF values were extracted using the GROMACS simulation package. Solvent accessible surface area (SASA) was computed using the implementation in GROMACS gmx sasa. The fraction of native contacts (Q2) was calculated from the last frame of the trajectory using the Python module MDAnalysis 2.2.0 (Gowers et al. 2016; Michaud‐Agrawal et al. 2011). Contacts were defined as pairs of residues with an alpha carbon distance of 8 Å or less. For the calculation of the DSSP (Kabsch and Sander 1983) and the solvent accessible surface area (Shrake and Rupley 1973) for the analysis of the protein UniprotID:Q12072 python package *MDTraj* 1.9.7 (McGibbon et al. 2015) was used. Dynamics were analyzed using VMD 1.9.4 and ChimeraX 1.4 (Goddard et al. 2018; Meng et al. 2006; Pettersen et al. 2021). The structural images shown in Figure 3 were made with VMD. VMD is developed with NIH support by the Theoretical and Computational Biophysics group at the Beckman Institute, University of Illinois at Urbana‐Champaign.

### 4.15 Partial correlations with nucleotide features

For both the model predictions and residuals, separately, we computed partial correlations with mRNA folding strength (mF) and tRNA adaptation index (tAI). The former was taken as the geometric mean of PARS score along the mRNA sequence, as provided in Kertesz et al. (2010), and the latter obtained from Tuller et al. (2010b). Partial correlation between a variable *X* and *Y* is defined as their correlation after linearly removing the effect of a set of controlling variables ***Z***. This was computed as the correlation of the residuals of *X* ~ ***Z*** and *Y* ~ ***Z***, using the *partial_corr* method with Pearson correlation in the Pingouin 0.5.4 Python package (Vallat 2018). The results in Figure S11a show partial correlation between predictions and mF while controlling for tAI, and between predictions and tAI while controlling for mF. Similarly, results are shown substituting predictions with residuals (Figure S11b) and actual protein abundance values (Figure S11c).

### 4.16 Proteomics analysis

The *S. cerevisiae* IOC2 knockout strain (*ioc2*Δ::*kanMX*) in the BY4741 (MATa *his3*Δ1 *leu2*Δ0 *met15*Δ0 *ura3*Δ0) background was requested from the Yeast Knockout (YKO) Collection (Winzeler 1999) in Gothenburg University and used for genomic engineering in the following procedures. Predicted mutant (UniprotID: Q12072) DNA sequences flanking with 90 bp overlap to the specific genome sites on both ends were ordered as gene fragments from either TWIST Bioscience (www.twistbioscience.com). The mutant DNA sequence was designed to not change original wildtype codons to minimally affect the translation. The predicted mutated amino acids were substituted using the most frequent corresponding codon.

To replace the *kanMX* gene (Winzeler 1999) with the mutant gene in the genome, a gRNA plasmid targeting *kanMX* was constructed based on an All‐In‐One plasmid pML104 (Laughery et al. 2015). The 20 bp gRNA sequence targeting at the *kanMX* gene (GCCGCGATTAAATTCCAACA) was designed with the CRISPR tool in Benchling (https://benchling.com). Primer sets pFA6‐KanMX 488–507 FWD/pML_F and pFA6‐KanMX 488–507 REV/f1 ori_R (Table S11) were used to amplify pML104 into 2 fragments pML104.part1 and pML104.part2 with 20 bp homologous sequences on both ends and gRNA sequence integrated in the pFA6‐KanMX 488–507 FWD/pFA6‐KanMX 488–507 REV primers. pML104.part1 and pML104.part2 were ligated into a circular plasmid named as pML104.gRNA_kanMX by Gibson Assembly (Gibson et al. 2009) and was sequence‐verified by Eurofins (https://www.eurofins.com/) with M13R primer (Table S11). pML104.gRNA_kanMX and mutant gene was transformed into a knockout strain with PEG/LiAc method (Gietz 2014) and selected on synthetic minimal medium without uracil (SD‐URA) plates. Colonies were verified with PCR using the primer set YLR095C_F/YLR095C_R (Table S1), and the amplified fragments were sequence‐verified by Eurofins (https://www.eurofins.com/) with YLR095C_F/YLR095C_R primer set. SD medium supplemented with 5‐fluoroorotic acid (SD + 5‐FOA) (Boeke et al. 1984) was used to select colonies for loss of pML104.gRNA_kanMX.

Recombinant colonies without plasmids and the wild‐type BY4741 colony were picked into the YPD medium. After overnight growth, 1% was inoculated into 1.5 mL YPD medium in a 48‐well flower plate (M2P labs), and each sample had triplicates. The 48 well flower plates were cultured in 30°C, 1200 rpm for either around 10 h in a Biolector (M2P labs) until the cell growth reached the mid‐exponential phase or 24 h until the cell growth reached the stationary phase. One milliliter cells from both phases were collected and washed with MilliQ water once. After centrifugation, the supernatant was removed, and cell pellets were kept at −80°C until sent to perform proteomics analysis at High Throughput Mass Spectrometry Core Facility, Charité (Berlin, Germany). The data‐independent acquisition was performed using the TimsTOF PRO mass spectrometer (Bruker) coupled to the UltiMate 3000 RSL (Thermo). The peptides were separated using the Waters ACQUITY UPLC HSST3 1.8 μm column at 40°C using a linear gradient ramping from 2% B to 40% B in 30 min (Buffer A: 0.1% FA; Buffer B: ACN/0.1% FA) at a flow rate of 5 μL/min. The column was washed by an increase in 1 min to 80% and kept by 6 min. In the following 0.6 min, the composition of B buffer was changed to 2%, and the column was equilibrated for 3 min. For MS calibration of ion mobility dimension, three ions of Agilent ESI‐Low Tuning Mix ions were selected (m/z [Th], 1/𝐾0 [Th]: 622.0289, 0.9848; 922.0097, 1.1895; 1221.9906, 1.3820). The dia‐PASEF windows scheme was ranging in dimension m/z from 400 to 1200 and in dimension 1/𝐾0 0.6–1.43, with 32 × 25 Th windows with Ramp Time 100 ms. Data quantification was performed using the DIA‐NN 1.8 software (Demichev et al. 2020), using library‐free mode. Q12072 protein's expression analysis in exponential and stationary phases (Data S1) was carried out using only the peptides that were detected in both growth phases in mutant and wildtypes correspondingly, that is, the protein changes are calculated as fold‐changes of corresponding Q12072 measured peptides in each strain. For the expression experiment, three biological replicates from mutant and wildtype were analyzed in each growth phase. The raw mass spectrometry data have been deposited to the ProteomeXchange Consortium via the PRIDE partner repository (Perez‐Riverol et al. 2019) with the dataset identifier PXD053435.

### 4.17 Statistical analyses

All statistical analyses were performed using the Python (3.9) package Scipy 1.8.1 (Virtanen et al. 2020) and R 4.2.0. For data manipulation and visualization, we used pandas 1.4.0 (The Pandas Development Team 2023) seaborn 0.12.2, (Waskom 2021) scikit‐learn 0.24.2 (Pedregosa et al. 2011), and the R tidyverse 2.0.0 (Wickham et al. 2019) package collection. Hypothesis testing was performed using the nonparametric Wilcoxon Rank Sum test unless indicated otherwise.

## AUTHOR CONTRIBUTIONS

**Filip Buric:** Conceptualization; methodology; software; data curation; investigation; validation; formal analysis; visualization; project administration; writing – original draft; writing – review and editing. **Sandra Viknander:** Conceptualization; methodology; software; data curation; investigation; validation; formal analysis; visualization; writing – original draft; writing – review and editing. **Xiaozhi Fu:** Validation; investigation; data curation. **Oliver Lemke:** Formal analysis; writing – original draft; visualization. **Oriol Gracia Carmona:** Investigation; methodology. **Jan Zrimec:** Conceptualization; methodology; software; investigation; formal analysis. **Lukasz Szyrwiel:** Investigation; validation. **Michael Muelleder:** Investigation; validation. **Markus Ralser:** Investigation; resources; writing – review and editing; funding acquisition. **Aleksej Zelezniak:** Conceptualization; investigation; funding acquisition; writing – original draft; writing – review and editing; visualization; validation; software; formal analysis; project administration; data curation; supervision; resources.

## CONFLICT OF INTEREST STATEMENT

M.R. and A.Z. are a co‐founders of Eliptica Limited. All other authors declare no competing interests.

## Supporting information


**Data S1.** Supporting Information.

## Data Availability

Scripts, training parameters, and software versions are provided in the following repository: https://github.com/fburic/protein-mgem. The models and data required to reproduce figures are stored in the following Zenodo record: https://zenodo.org/doi/10.5281/zenodo.8377126.
