# Supplementary material for: Amino acid sequence encodes protein abundance shaped by protein stability at reduced synthesis cost
Source: Protein Sci. 2024 Dec 12;34(1):e5239. doi: 10.1002/pro.5239 (PMC11635393; doi:10.1002/pro.5239)
Supplement: Supplementary file 1 — Data S1. Supporting Information. [file PRO-34-e5239-s001.docx]

**Amino acid sequence encodes protein abundance shaped by protein stability at reduced synthesis cost.**

Filip Buric^1*^, Sandra Viknander^1*^, Xiaozhi Fu^1^, Oliver Lemke^2^, Oriol Gracia Carmona^3,4^, Jan Zrimec^1,5^, Lukasz Szyrwiel^2^, Michael Muelleder^6^, Markus Ralser^2^, Aleksej Zelezniak^1,3,7✝^

1 - Department of Biology and Biological Engineering, Chalmers University of Technology, Kemivägen 10, SE-412 96, Gothenburg, Sweden

2 - Department of Biochemistry, Charité – Universitätsmedizin Berlin, 10117 Berlin, Germany

3 - Randall Centre for Cell & Molecular Biophysics, King’s College London, New Hunt's House, Guy's Campus, SE1 1UL London, UK

4 - Institute of Structural and Molecular Biology, University College London, WC1E 6BT London, UK

5 - Department of Biotechnology and Systems Biology, National Institute of Biology, Večna pot 111, SI1000 Ljubljana, Slovenia

6 - Core Facility High Throughput Mass Spectrometry, Charité – Universitätsmedizin Berlin, 10117 Berlin, Germany

7 - Institute of Biotechnology, Life Sciences Centre, Vilnius University, Sauletekio al. 7, LT10257 Vilnius, Lithuania

*These authors contributed equally

^✝^corresponding author (email: aleksej.zelezniak@chalmers.se)

**Keywords**: proteome, protein sequence, protein expression, protein engineering, protein stability, deep learning, language models, explainable machine learning, molecular dynamics

Contents

[Supplementary Figures 2](#_4wg09no44nzv)

[Supplementary Note 18](#_yu927v2cykct)

[Supplementary Tables 20](#_5qakqbjou97f)

[Supplementary References 30](#_ojz6hc7rhnsk)

##

# Supplementary Figures


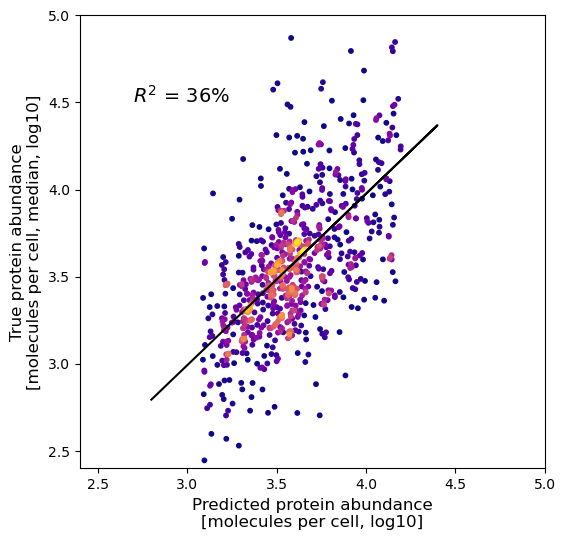

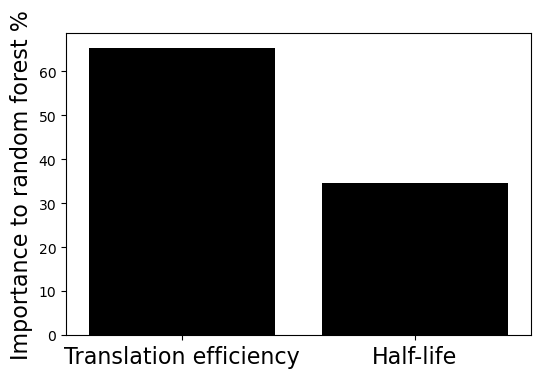


**Figure S1. Translation efficiency and protein half-life predict protein abundance.** Performance (on a hold-out test set) of a random forest model with translation efficiency and half-life as input factors, coloured by density. **Inset:** Importance of the two factors (65% and 35%, respectively) to the random forest model.

#
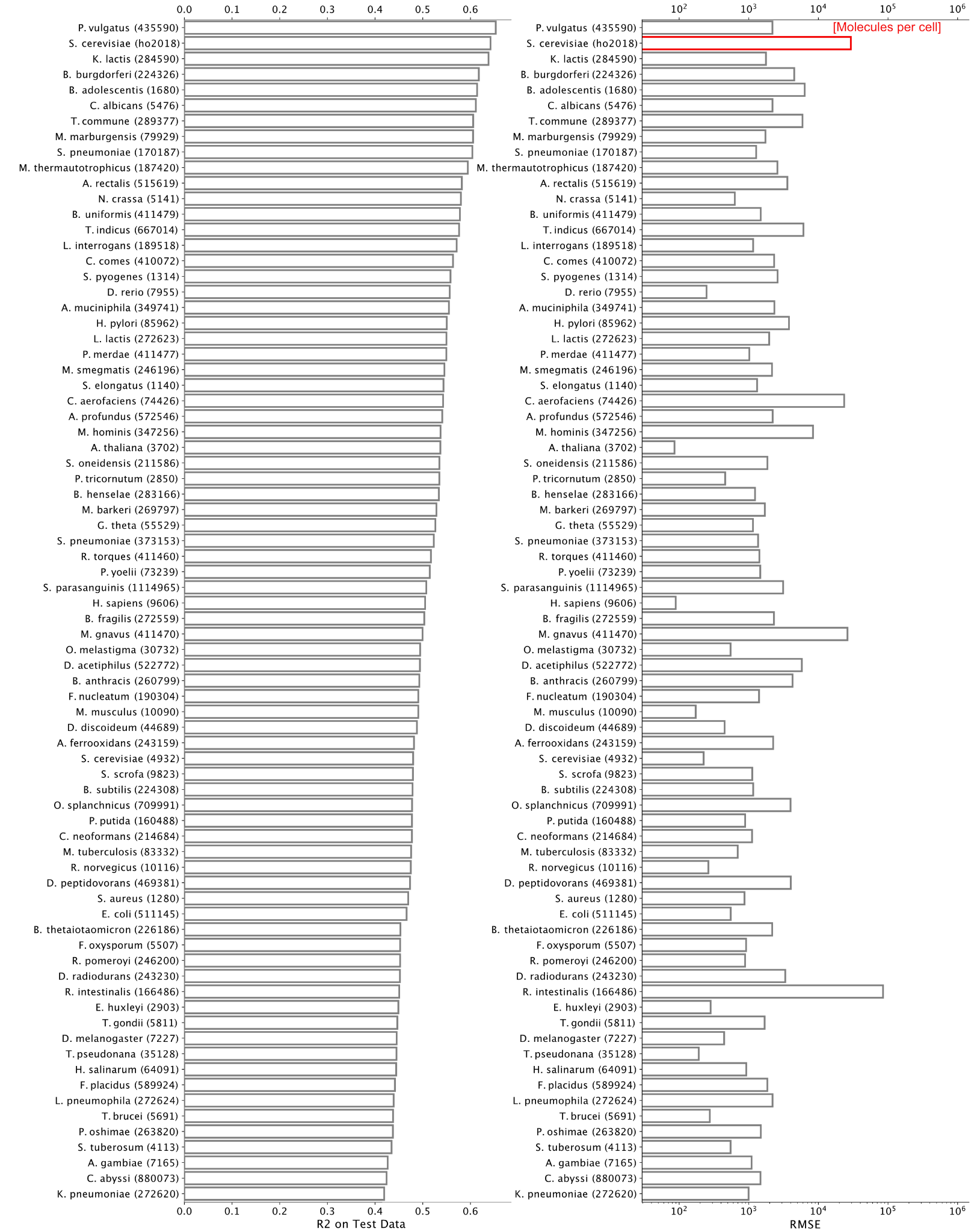

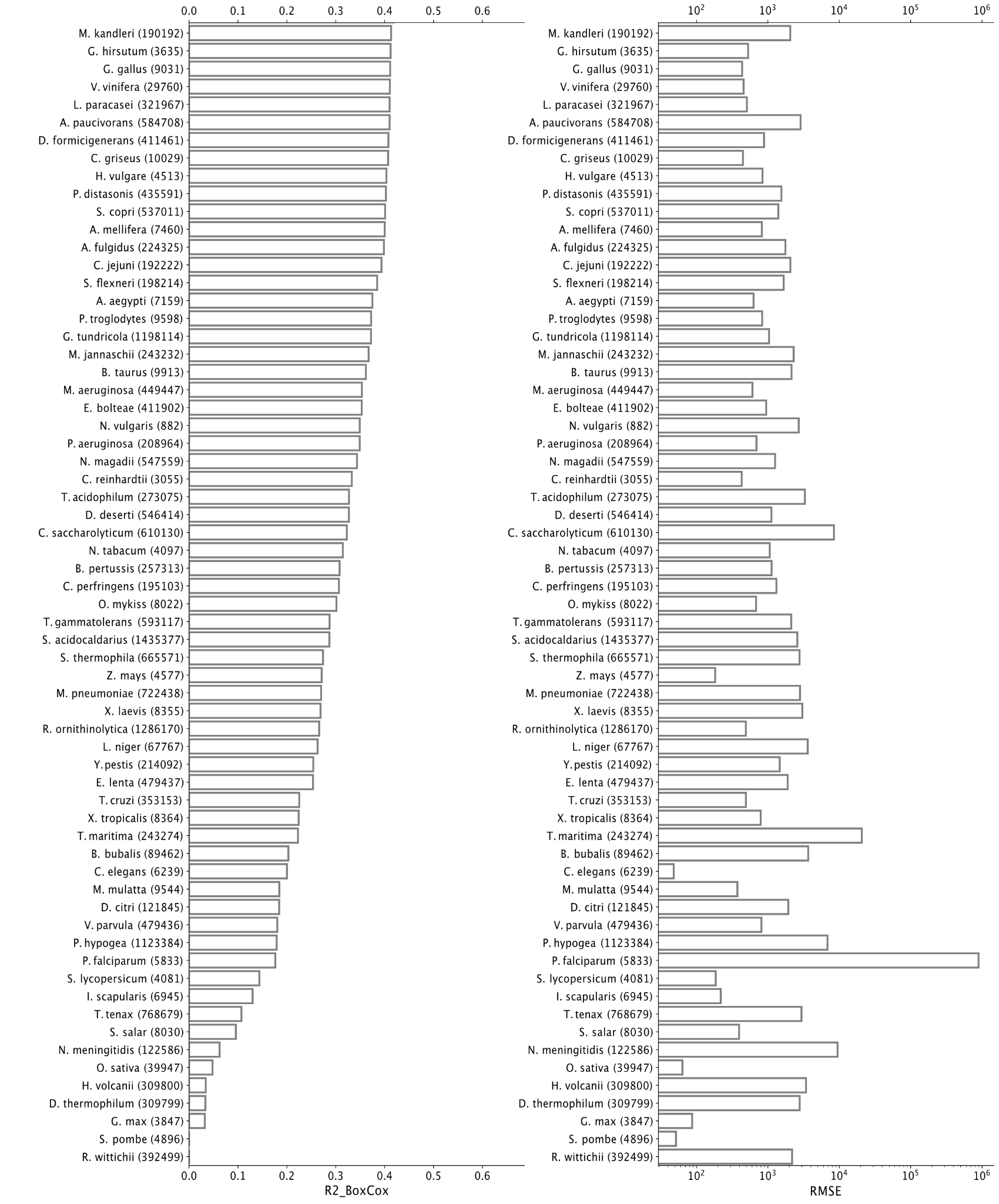


**Figure S2. Model performance for 139 of the organisms in PaxDB** [**(Huang et al. 2023)**](https://paperpile.com/c/9fsz4a/iqqU) **as well as Ho2018** [**(Ho et al. 2018)**](https://paperpile.com/c/9fsz4a/8pNWk) **data set, as the coefficient of determination (left) and root mean squared error (right).** The models were trained on median abundance values across all experiment data sets for a given organism. All models were evaluated on test sets for medians (bars). The coefficient of determination (R^2^) was computed on Box-Cox-transformed data. The root mean squared error was computed for PaxDb, in units of parts per million (PPM) and number of molecules per cell for the Ho2018 data.


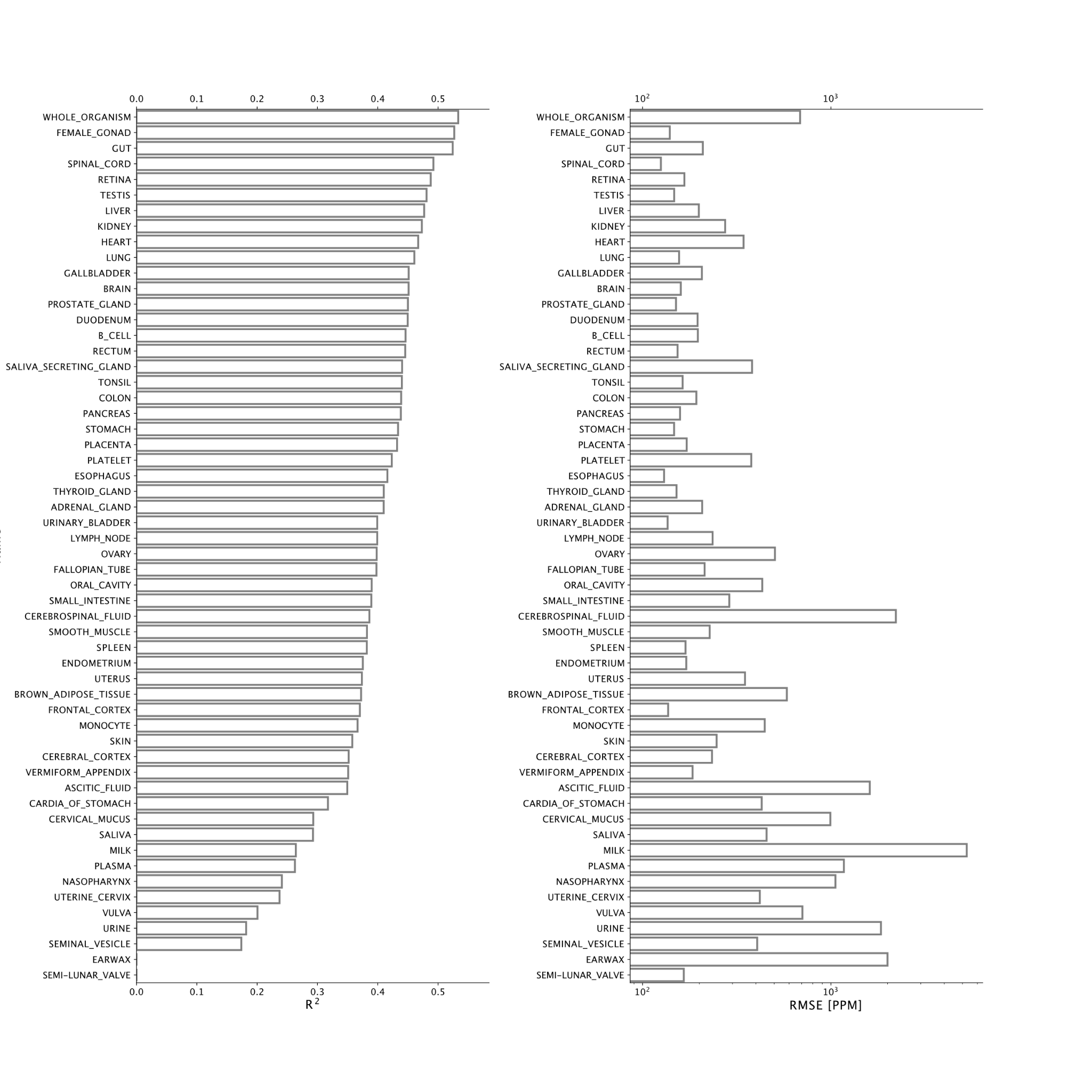


**Figure S3. Model performance for 57 tissues from H. Sapiens data set in PaxDB**[**(Huang et al. 2023)**](https://paperpile.com/c/9fsz4a/iqqU)**, as coefficient of determination (left) and root mean squared error (right).** The models were trained on median abundance values across experiments from the different tissue types. All models were evaluated on test sets for medians. The coefficient of determination (R^2^) was computed on Box-Cox-transformed data. The root mean squared error was computed for PaxDb, in units of parts per million (PPM).

**
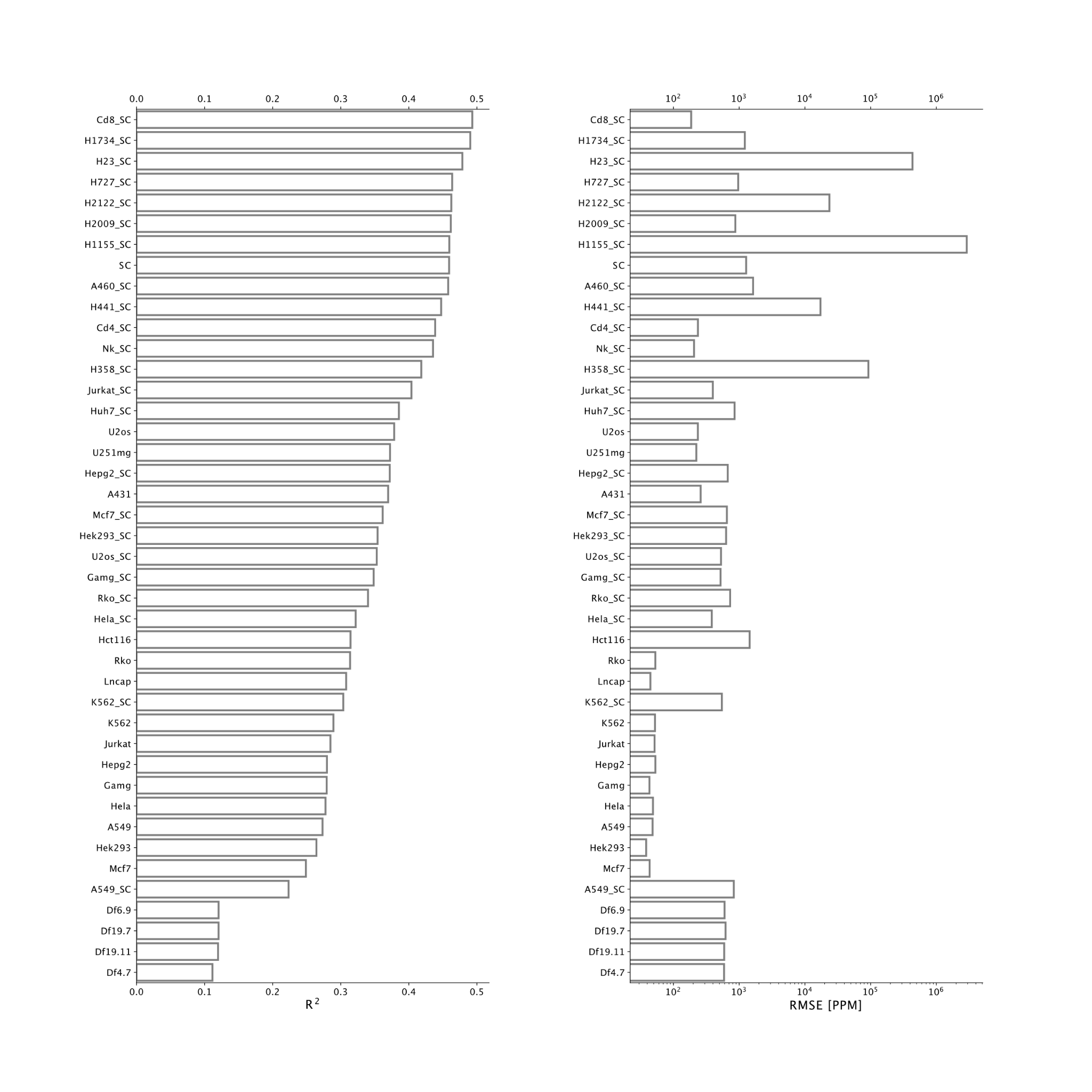
**

**Figure S4. Model performance for 42 H. Sapiens cell lines data sets in PaxDB** [**(Huang et al. 2023)**](https://paperpile.com/c/9fsz4a/iqqU)**, as coefficient of determination (left) and root mean squared error (right).** The models were trained on median abundance values across experiments from the different tissue types. All models were evaluated on test sets for medians. The coefficient of determination (R^2^) was computed on Box-Cox-transformed data. The root mean squared error was computed for PaxDb, in units of parts per million (PPM).


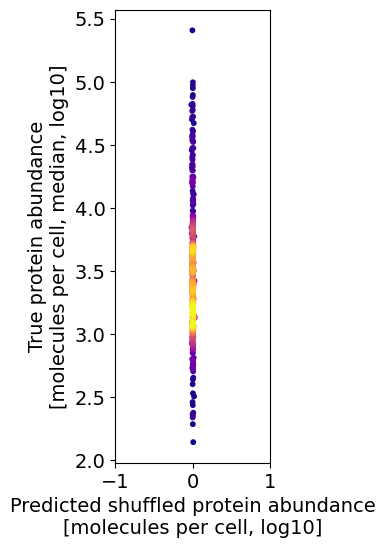


**Figure S5. Random prediction control of our Transformer model using shuffled versions of the test set sequences.** The poor performance on randomized input, predicting effectively a single value, demonstrates that the model has learned sequence structure and not amino acid frequencies.

| **A**  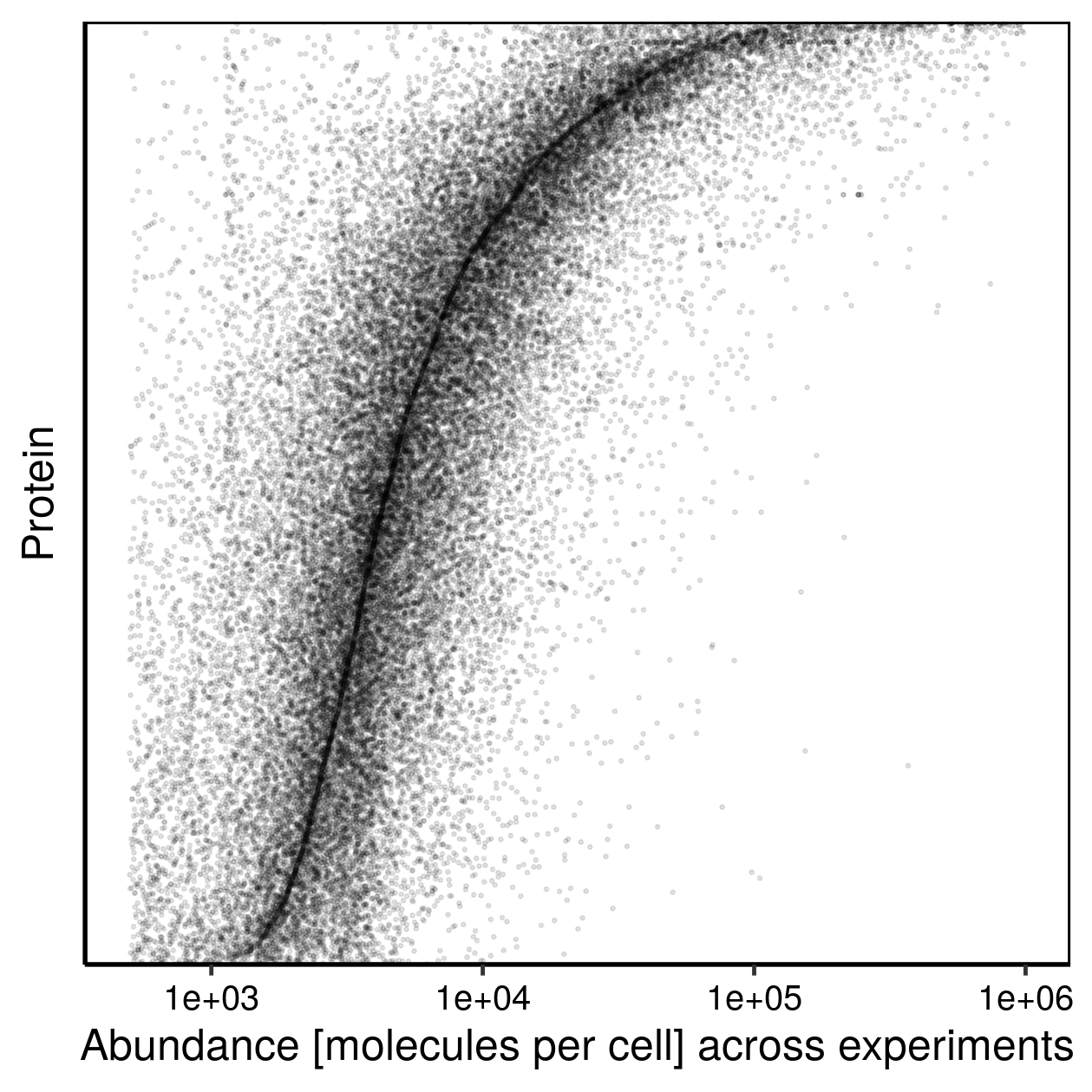 | **B**  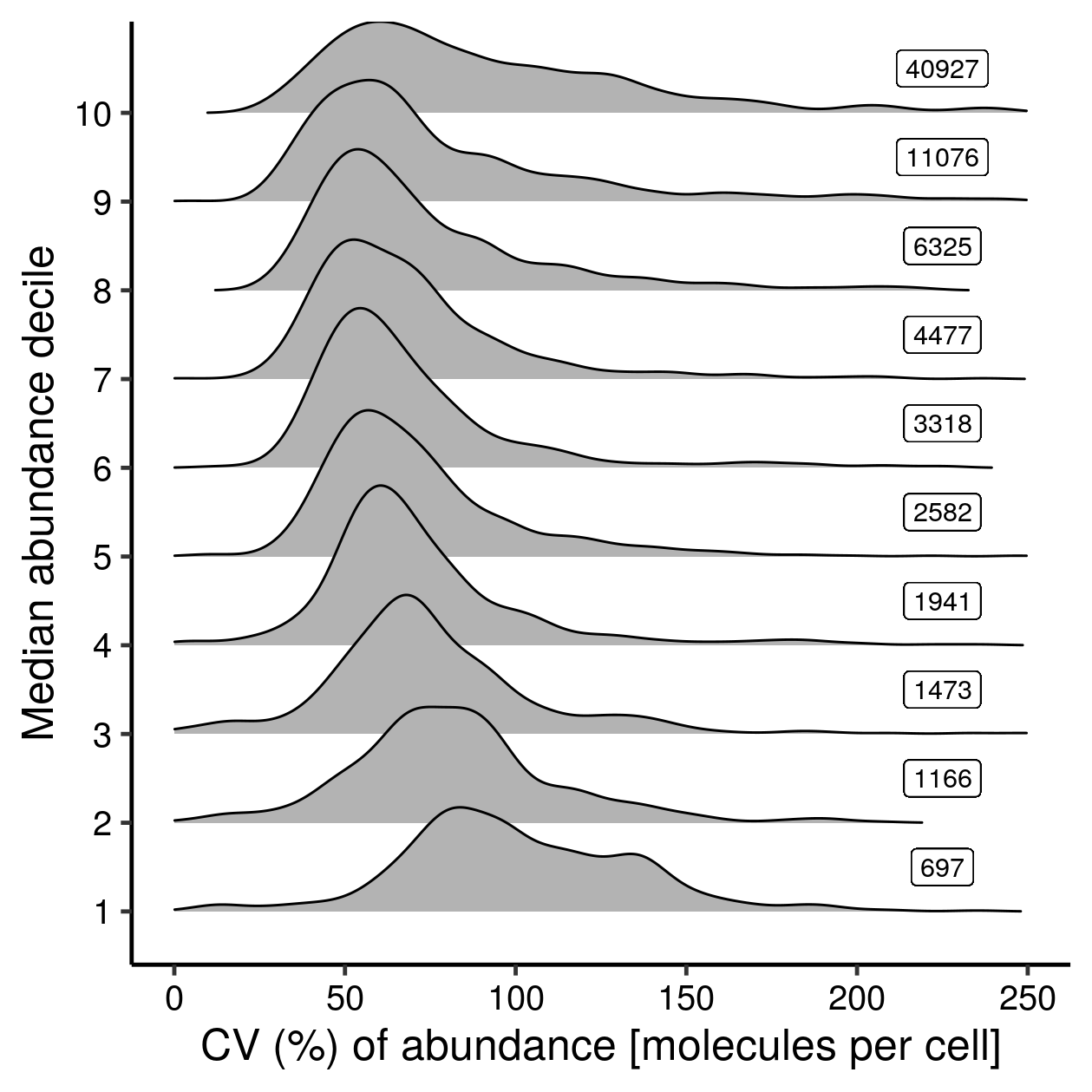 |
| --- | --- |
| **C**  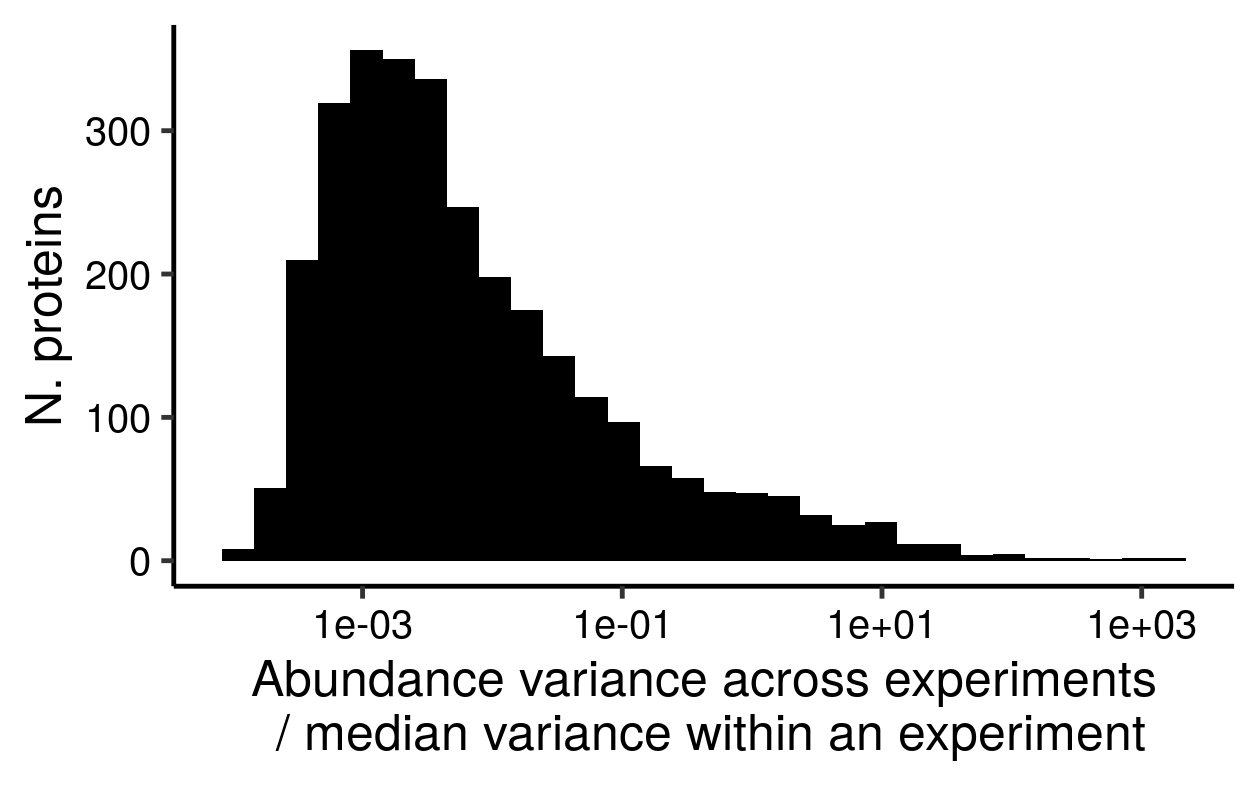 | **D**  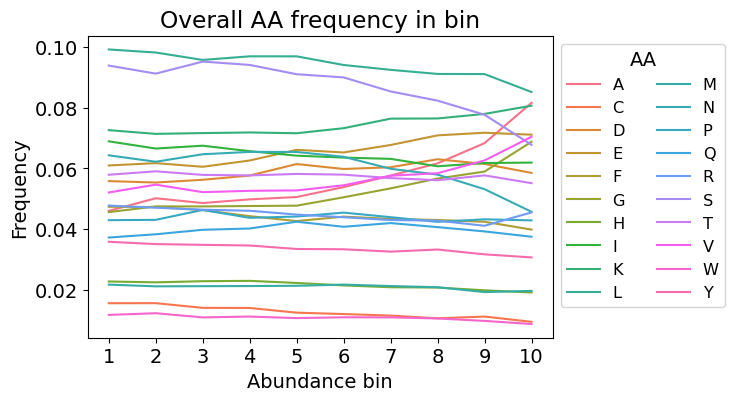 |

**Figure S6.** **Protein abundance and amino acid frequency have constrained variance.**

**A)** Protein abundance variation from data in [(Ho, Baryshnikova, and Brown 2018)](https://paperpile.com/c/9fsz4a/8pNWk)), showing the narrow variation across experiments for each protein (proteins with fewer than 10 experiment values were discarded)**. B)** Distribution of the coefficient of variation of protein abundance across experiments, grouped by abundance decile (right labels indicate median abundance for each decile). **C)** Distribution of ratios between the variance of protein abundance across experiments and the median of variances within each experiment. **D)** Amino acid frequencies across abundance deciles.


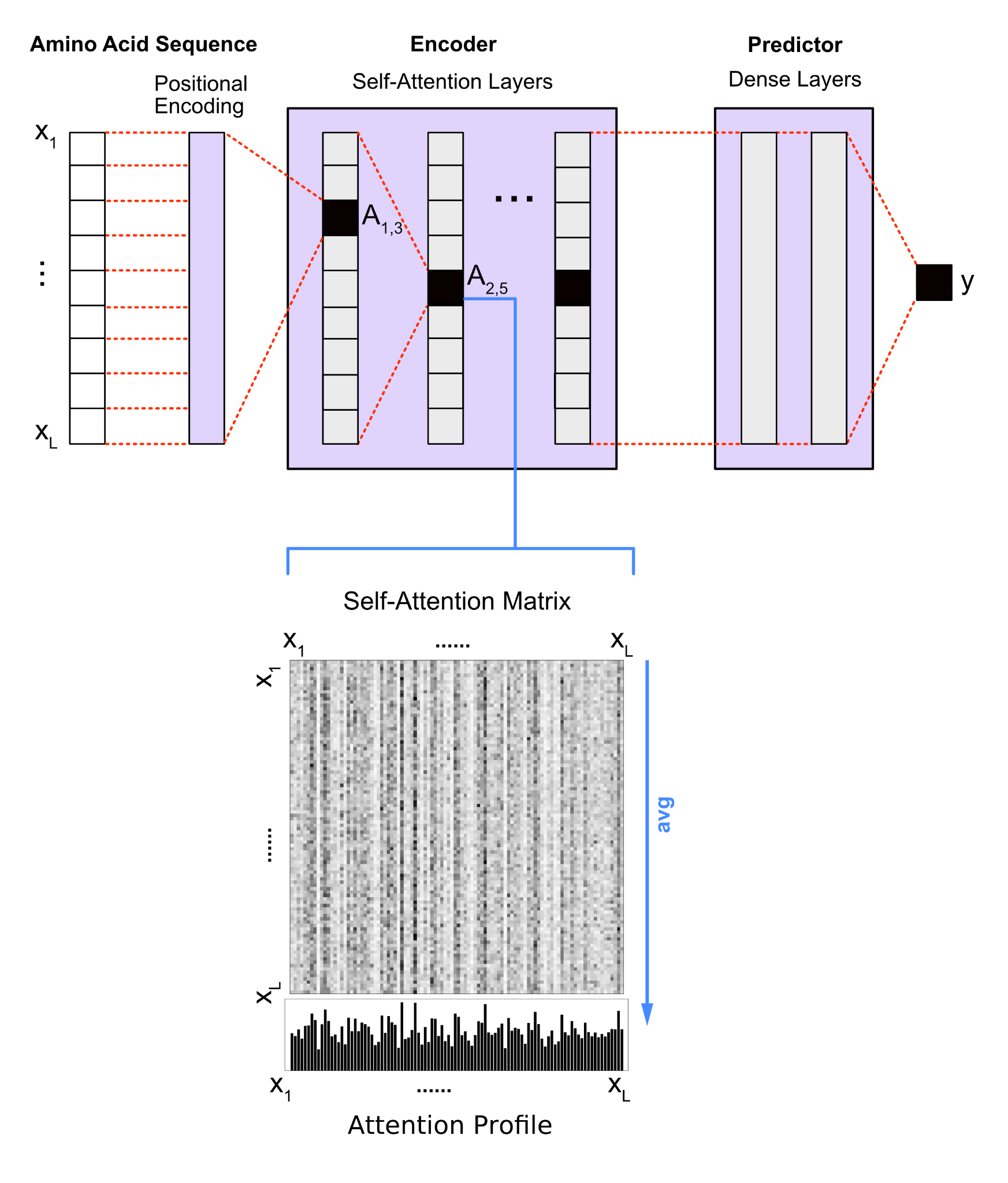


**Figure S7. Diagram of our Transformer (BERT) neural network (top) and examples of a self-attention matrix and derived attention profile (bottom).** Each head A_i,j_ in an attention layer i outputs an attention matrix consisting of directional association weights between pairs of residues in the amino acid sequence (X, of length L), normalized as a percentage (across the entire matrix). The one-dimensional attention profiles used in this study were obtained by averaging along the “attends-to” axis, as the “attended-by” variation is generally more informative. A high value in a profile position signifies that the position “receives a lot of attention” from all other positions. Note that for a given sequence, one obtains N x H such matrices, for N attention layers and H heads.


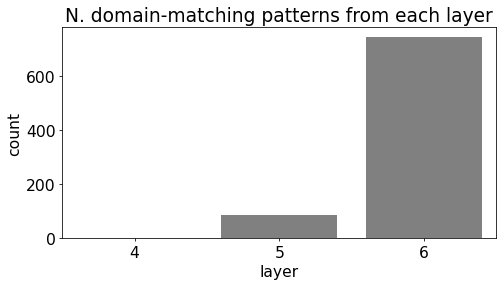


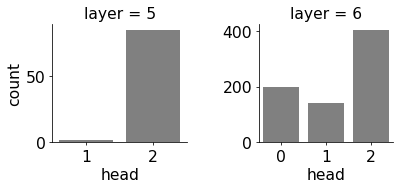


**Figure S8. Number of attention patterns matching protein domains, by layer and head.** The counts show the number of attention patterns that matched a protein domain in more than 30% of its residues. These patterns were obtained almost exclusively from the attention matrices of only a few heads, in the deeper attention layers of the Transformer network (layers are numbered from 0 to 7).


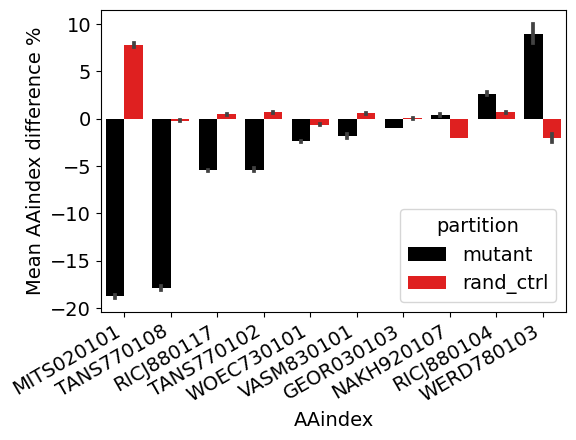

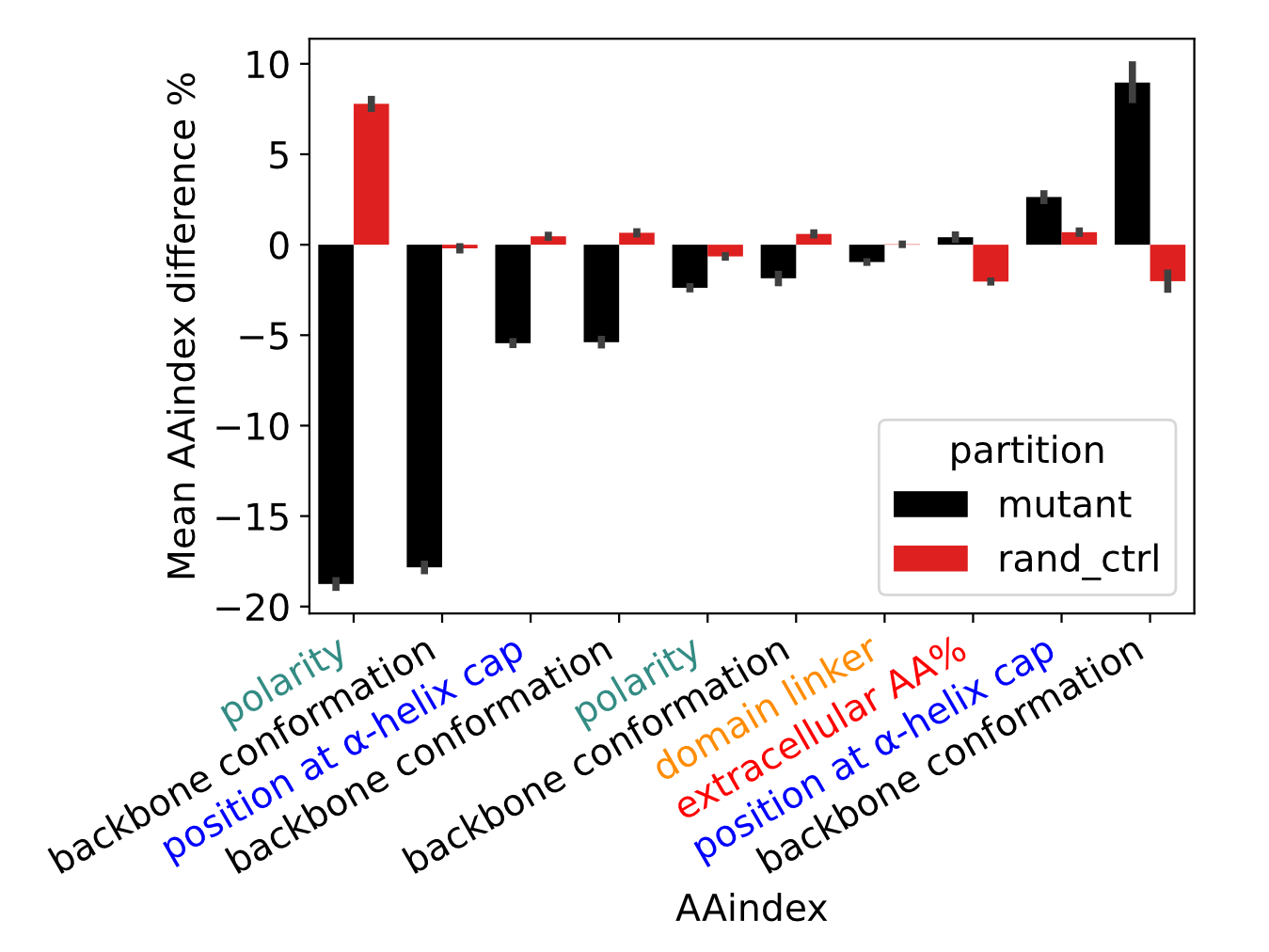


**Figure S9. The average sequence AAindex value of mutants obtained with MGEM show significant shifts compared with random control (paired t-test, p-value < 1e-308).** **Top**: AA indices are labeled by their AAindex IDs. **Bottom**: The indices are labeled by their type.


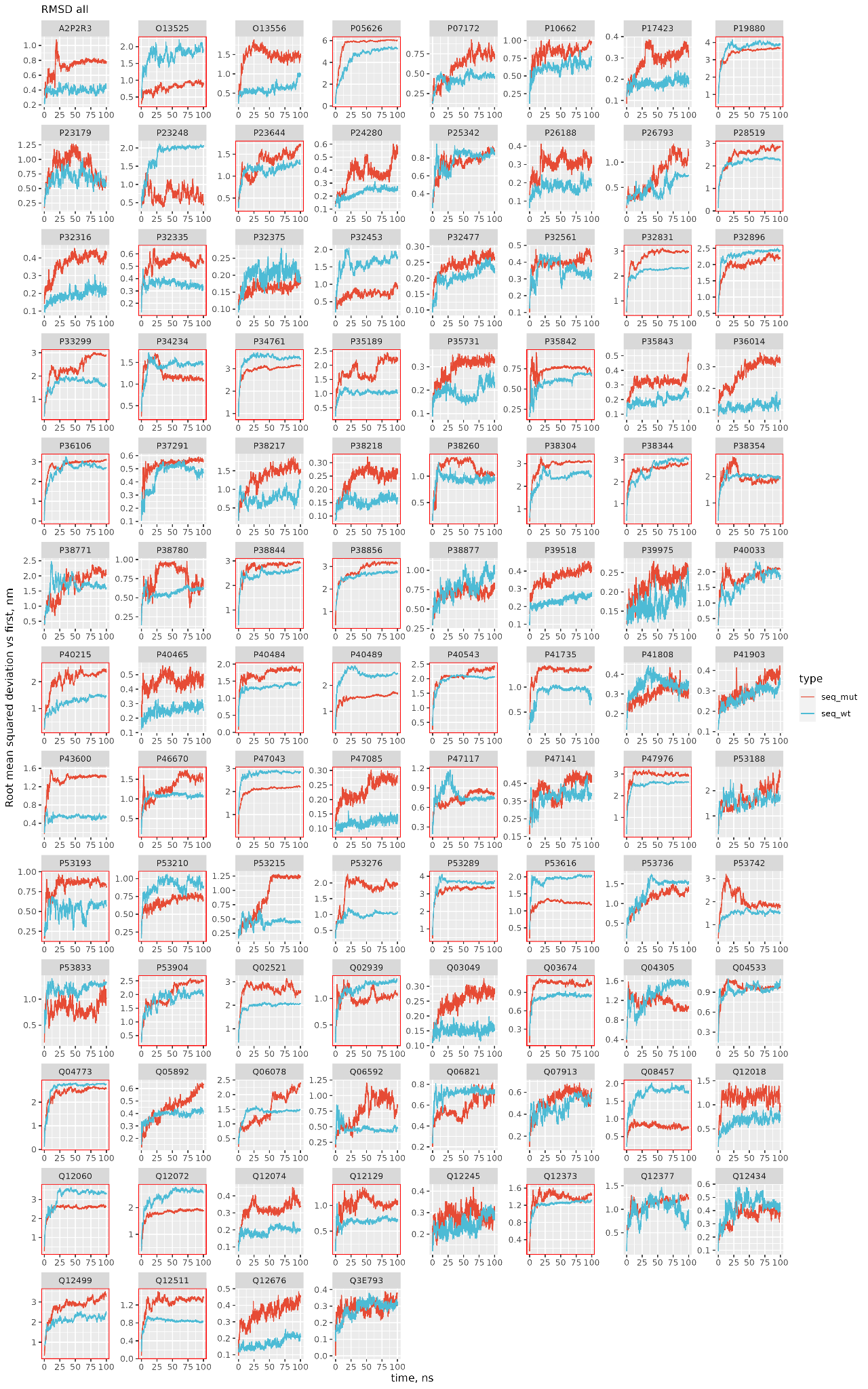


**Figure S10.** **Root mean squared deviation from first frame over 100 ns of MD simulations.** Highlighted are converged simulations where last quarter is within 10% of deviations (see main text Methods M12).

**
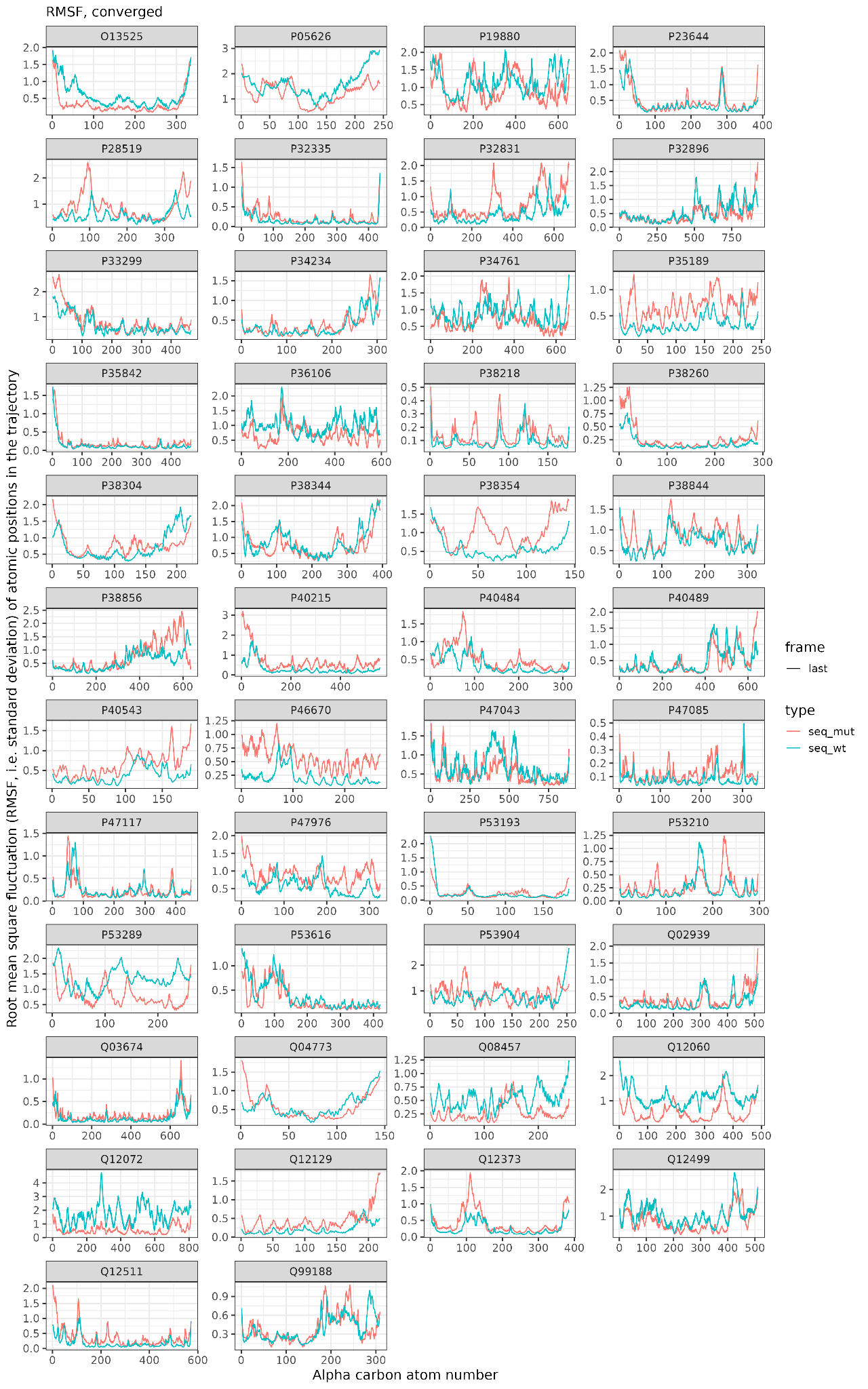
**

**Figure S11**. **Root mean square fluctuations over 100 ns of MD simulations referenced to the last frame.** Showing only converged simulations.


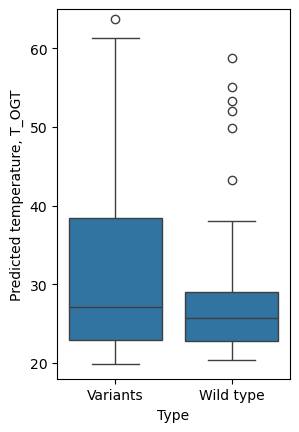


**Figure S12.** **Predicted** **organism growth temperature (T_OGT_) in MGEM mutants with increased predicted abundance (Variants) vs wild-type sequences**. Predictions were carried out using the DeepET model [(Li et al. 2022)](https://paperpile.com/c/9fsz4a/09Pm).


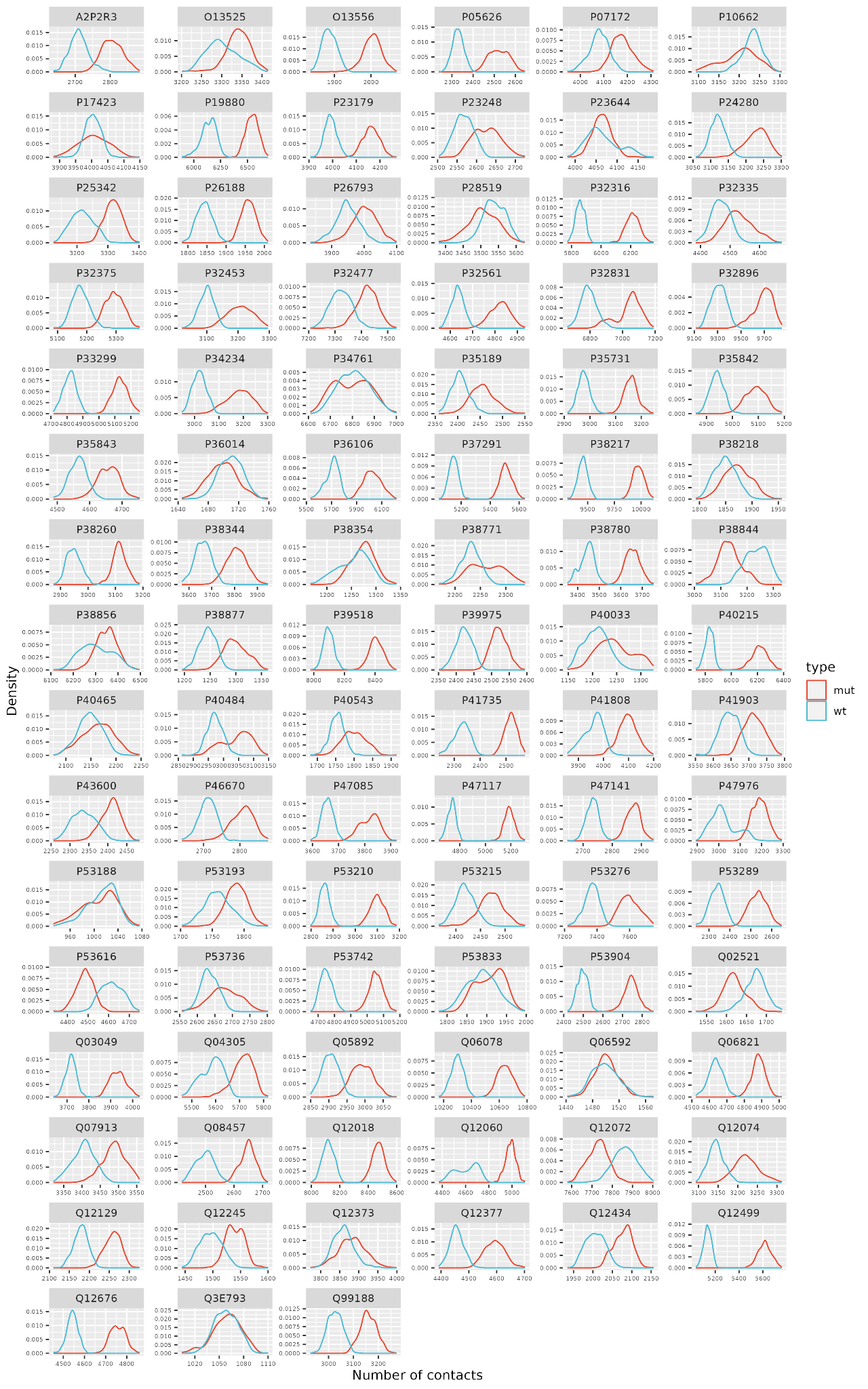


**Figure S13. Contacts Number contacts over simulation trajectory.** The contact is defined within 8Å distance between alpha carbons (see Methods M14).


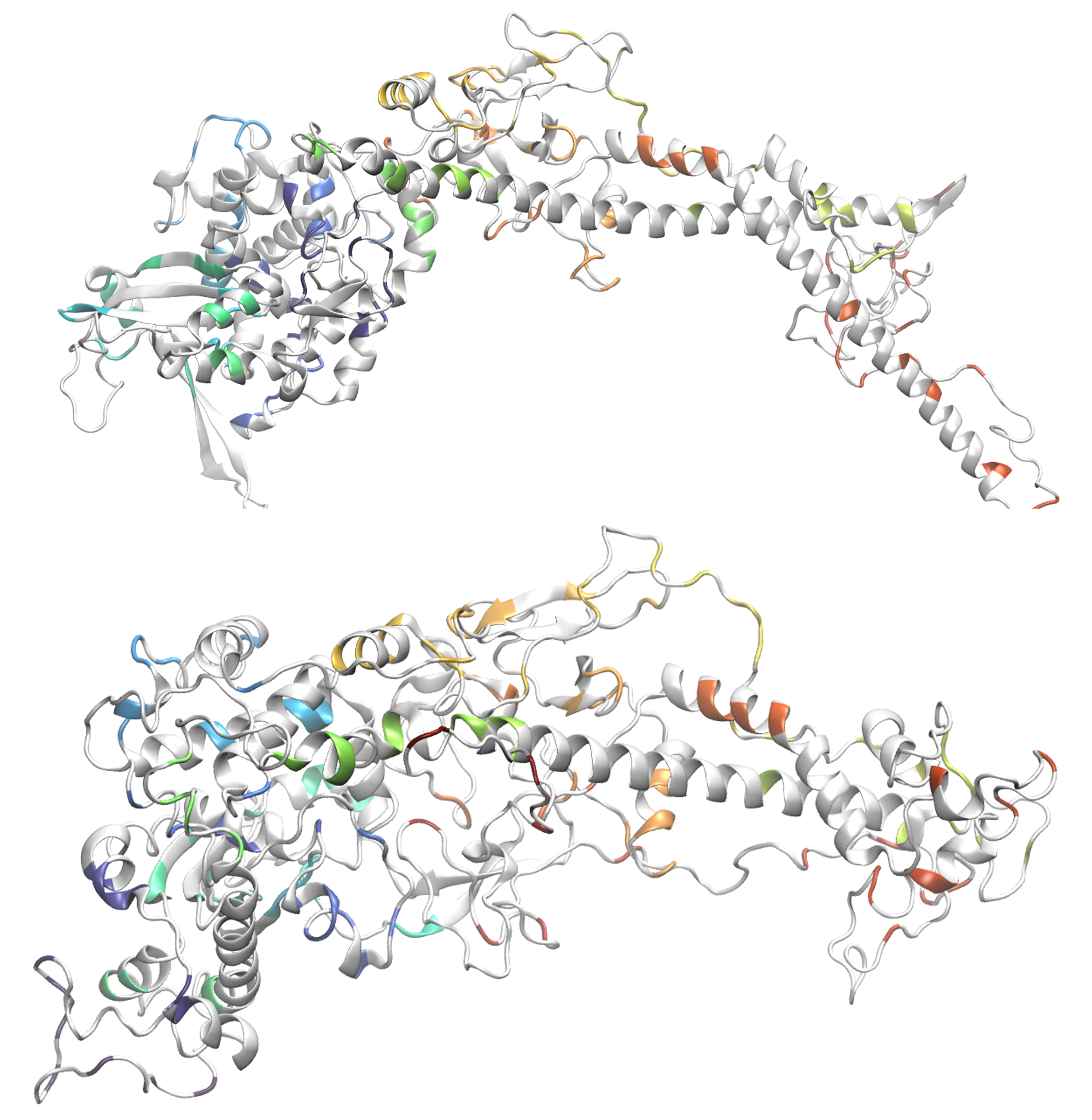


**Figure S14. IOC2 yeast protein.** Mutated positions are highlighted in colors. The top panel denotes the last frame of the simulation of the wild type, the bottom panel of the mutant. The coloring is according to the amino acid index as shown in Figure 3F in the main text.

| **A**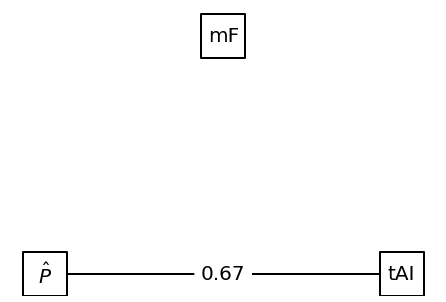 | **B**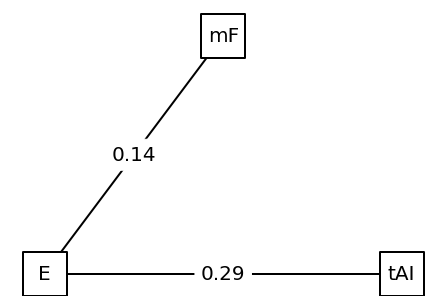 |
| --- | --- |
| **C**  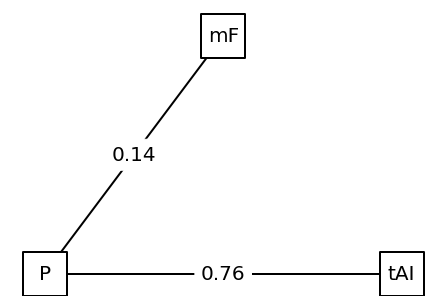 | |

**Figure S15. Partial Pearson correlations of model predictions (**$\hat{\boldsymbol{P}}$**), residuals (E), and target abundance values (P).** Values computed over the n = 239 intersection of the model’s test set and available mRNA folding strength (mF) and tRNA adaptation index (tAI) data. Abundance values are Box-Cox-transformed.

**A)** Partial correlations between $\hat{P}$ and mF, controlling for tAI (r=-0.0097, p-value = 0.88, discarded), and between $\hat{P}$ and tAI, controlling for mF (p-value < 1e-32) **B)** Partial correlations between *E* and mF, controlling for tAI (p-value < 1e-5), and between *E* and tAI, controlling for mF (p-value < 1e-5).

**C)** Partial correlations between *P* and mF, controlling for tAI (p-value < 0.05), and between *P* and tAI, controlling for mF (p-value < 1e-45).

# Supplementary Note

Following the molecular dynamics results (Figure 3A,F) to test the feasibility of whether the abundance-increasing mutations could affect expression *in vivo,* we performed an experiment in *S. cerevisiae.*  Namely, we genetically replaced the open reading frame of the native WT IOC2 protein – the protein with the highest observed RMSF perturbations – with the synthetic variant obtained by mutating 20% of its residues using MGEM (Methods M11). Using a liquid chromatography-coupled mass spectrometer (LC-MS) in data-independent acquisition mode [^95,96^](https://paperpile.com/c/kEGQ1w/JPsVK+9YC34), we monitored the IOC2 expression in E and S growth phases (Methods M16), growing yeast in triplicates to compare the WT and mutant variant (n = 3 per group). As a proxy for protein expression change, we compared protein levels between exponential (E) and stationary (S) phases within the same strain. This is crucial from a technical perspective as we are introducing 20% mutated residues into the WT sequence, which means the peptide tryptic digest results in entirely different fragment ion spectra between mutant and WT, thus making quantitative comparisons between strains infeasible, as we are measuring different analytes. As such, we observed that the quantified IOC2 peptides of the mutant variant were, on average, ~50% more highly expressed (Supplementary Note Figure 1) between the S and E phases in comparison to the WT control (Methods 16), demonstrating that the mutant version of IOC2 extended the expression into the stationary phase in contrast to the wild type. While the conclusions drawn from a single experiment do not prove that expression changes are related to proteins’ stability, nevertheless, the results support the experimental feasibility of the MGEM procedure and the observed data aligns with the overall result from our MD experiments, namely that an increase in rigid conformations is accompanied by an increase of prolonged expression of protein levels in the stationary phase.


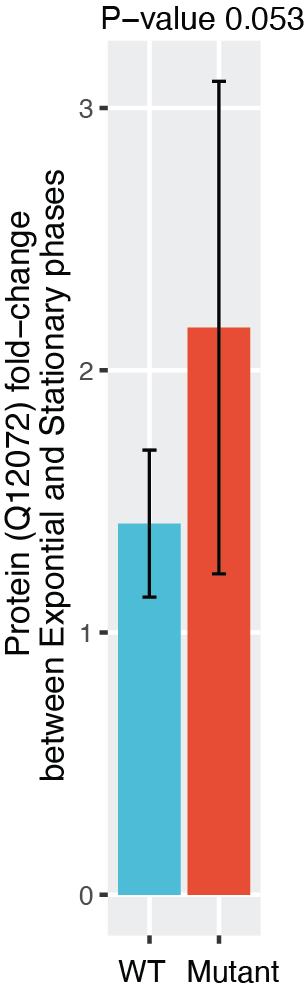


**Supplementary Note Figure.** Expression level fold-changes of (UniprotID: Q12072) IOC2-related peptides between exponential and stationary phases in WT and mutant strains. The experiment was performed in biological triplicates (Methods M16).

#

# Supplementary Tables

**Table S1. Amino acid costs that correlate with attention profiles.** The maximum absolute Pearson correlation (with p-value < 1e-5) was chosen among all the attention profiles of a given sequence. The table separates the positively and negatively correlated sequence subpopulations, showing mean values and subpopulation counts.

| **Cost** | **Mean pos. corr.** | **Pos. count** | **Mean neg. corr.** | **Neg. count** | **Description** |
| --- | --- | --- | --- | --- | --- |
| yeast_car_rel | 0.322725 | 1855 | -0.328086 | 705 | Impact of rel. change of the AA requirement on the minimal intake of C (glucose) [(Barton et al. 2010)](https://paperpile.com/c/9fsz4a/neRHX) |
| craig_energy | 0.319014 | 1848 | -0.209371 | 684 | Energetic cost (avg. n. units of high energy P bonds and reducing H atoms required to produce the AA from glucose) [(Craig and Weber 1998)](https://paperpile.com/c/9fsz4a/qAZt) |
| wagner_resp | 0.309522 | 32 | -0.346841 | 4226 | Cost of synthesis under respiratory growth [(Wagner 2005)](https://paperpile.com/c/9fsz4a/d9jW) |
| wagner_ferm | 0.309046 | 66 | -0.329704 | 4057 | Cost of synthesis under fermentative growth [(Wagner 2005)](https://paperpile.com/c/9fsz4a/d9jW) |
| yeast_nit_abs | 0.248459 | 1833 | -0.272822 | 1165 | Impact of abs. change of the AA requirement on the minimal intake of N (ammonium) [(Barton et al. 2010)](https://paperpile.com/c/9fsz4a/neRHX) |
| akashi | 0.244109 | 33 | -0.286920 | 3341 | Energetic cost (avg. n. units of high energy P bonds and reducing H atoms) [(Akashi and Gojobori 2002)](https://paperpile.com/c/9fsz4a/oO51) |
| yeast_car_abs | 0.241446 | 121 | -0.289392 | 2797 | Impact of abs. change of the AA requirement on the minimal intake of C (glucose) [(Barton et al. 2010)](https://paperpile.com/c/9fsz4a/neRHX) |
| weight | 0.230809 | 1438 | -0.325723 | 1472 | Molecular weight of the amino acid (proxy for synthesis cost) [(Seligmann 2003)](https://paperpile.com/c/9fsz4a/5mGj) |
| yeast_nit_rel | 0.230152 | 1422 | -0.289971 | 4 | Impact of rel. change of the AA requirement on the minimal intake of N (ammonium) [(Barton et al. 2010)](https://paperpile.com/c/9fsz4a/neRHX) |
| craig_steps | N/A | 0 | -0.311740 | 3932 | The number of biosynthetic steps between central metabolism and the resulting AA [(Craig and Weber 1998)](https://paperpile.com/c/9fsz4a/qAZt) |

**Table S2. AAindex variables that correlate with attention profiles.** The maximum absolute Pearson correlation (with p-value < 1e-5) was chosen among all the attention profiles of a given sequence, for each profile. Shown are the mean correlation values across all proteins. AAindex values were fetched from [https://www.genome.jp/aaindex](https://www.genome.jp/aaindex/) (release 9.1 2006).

| **AAindex** | **Mean corr.** | **AAindex type** | **Publication** |
| --- | --- | --- | --- |
| RICJ880104 | 0.368029 | preference for position at α-helix cap | [(Richardson and Richardson 1988)](https://paperpile.com/c/9fsz4a/HglE) |
| WOEC730101 | 0.348570 | polarity | [(Woese 1973)](https://paperpile.com/c/9fsz4a/J1qc) |
| MITS020101 | 0.305923 | polarity | [(Mitaku, Hirokawa, and Tsuji 2002)](https://paperpile.com/c/9fsz4a/wTVi) |
| TANS770108 | 0.304256 | backbone conformation propensity | [(Tanaka and Scheraga 1977)](https://paperpile.com/c/9fsz4a/PvJ1) |
| VASM830101 | 0.301944 | backbone conformation propensity | [(Vasquez, Nemethy, and Scheraga 1983)](https://paperpile.com/c/9fsz4a/6RRq) |
| NAKH920107 | -0.119901 | extracellular AA% (measured in membrane proteins) | [(Nakashima and Nishikawa 1992)](https://paperpile.com/c/9fsz4a/xKxB) |
| RICJ880117 | -0.168564 | preference for position at α-helix cap | [(Richardson and Richardson 1988)](https://paperpile.com/c/9fsz4a/HglE) |
| GEOR030103 | -0.266115 | domain linker propensity | [(George and Heringa 2002)](https://paperpile.com/c/9fsz4a/mVHA) |
| TANS770102 | -0.337806 | backbone conformation propensity | [(Tanaka and Scheraga 1977)](https://paperpile.com/c/9fsz4a/PvJ1) |
| WERD780103 | -0.381838 | backbone conformation propensity | [(Wertz and Scheraga 1978)](https://paperpile.com/c/9fsz4a/IDe9) |

**Table S3. AAindex variables that correlate with attention profiles, separated in positively and negatively correlated protein subpopulations.** The maximum absolute Pearson correlation (with p-value < 1e-5) was chosen among all the attention profiles of a given sequence, for each profile. Shown are the mean correlation values across all proteins. AAindex values were fetched from [https://www.genome.jp/aaindex](https://www.genome.jp/aaindex/) (release 9.1 2006).

| **AAindex** | **Mean pos. corr.** | **Pos. count** | **Mean neg. corr.** | **Neg. count** | **AAindex type** |
| --- | --- | --- | --- | --- | --- |
| TANS770108 | 0.383633 | 3863 | -0.337209 | 482 | backbone conformation propensity |
| WERD780103 | 0.376552 | 8 | -0.383365 | 4359 | backbone conformation propensity |
| VASM830101 | 0.369531 | 3874 | -0.373924 | 386 | backbone conformation propensity |
| RICJ880104 | 0.369205 | 4018 | -0.231578 | 7 | preference for position at α-helix cap |
| NAKH920107 | 0.352343 | 1348 | -0.328829 | 3029 | extracellular AA%  (measured in membrane proteins) |
| WOEC730101 | 0.348830 | 4389 | -0.224522 | 1 | polarity |
| GEOR030103 | 0.319206 | 270 | -0.310555 | 3565 | domain linker propensity |
| MITS020101 | 0.310673 | 3740 | -0.245472 | 32 | polarity |
| RICJ880117 | 0.295534 | 1016 | -0.329225 | 2944 | preference for position at α-helix cap |

**Table S4.** **GO slim terms for *S. cerevisiae* proteins that have domains captured by the model attention mechanism.** The GO slim terms were mapped from the significant GO enrichment analysis terms (Holm-Bonferroni-corrected p-value < 0.05).

| **Biological Process** | **Molecular Funcion** | **Cellular Component** |
| --- | --- | --- |
| generation of precursor metabolites and energy  nucleobase-containing small molecule metabolic process  tRNA aminoacylation for protein translation  protein folding  response to chemical  translational elongation  chromatin organization  protein targeting  regulation of translation  sporulation  protein dephosphorylation  protein phosphorylation  monocarboxylic acid metabolic process  carbohydrate metabolic process | unfolded protein binding  transferase activity  lyase activity  ATP hydrolysis activity  kinase activity  ATPase-dependent activity  translation factor activity, RNA binding  cytoskeletal protein binding  GTPase activity  peptidase activity  glycosyltransferase activity  oxidoreductase activity  transmembrane transporter activity  hydrolase activity  DNA binding  phosphatase activity  ion binding  helicase activity  ligase activity | cytoplasm  cytoskeleton  mitochondrion  endoplasmic reticulum  membrane |

**Table S5. The leading 30% of protein sequences in the yeast proteome differs in composition from the overall sequence.** The amino acid counts of 4750 proteins were computed for the leading 30% region and for the entire sequence. These counts were compared using one-sided hypergeometric tests for enrichment and depletion, with a threshold p-value of 0.05.

| **Enriched AA** | **p-value** | **Depleted AA** | **p-value** |
| --- | --- | --- | --- |
| A | 9.679602e-04 | C | 3.849427e-15 |
| H | 2.059567e-03 | D | 3.104653e-08 |
| M | 5.330124e-76 | E | 1.043352e-49 |
| P | 7.746553e-22 | F | 1.895432e-15 |
| Q | 1.768194e-10 | G | 2.204858e-08 |
| R | 3.393047e-08 | I | 1.693833e-22 |
| S | 2.381427e-128 | K | 8.558290e-08 |
| T | 4.209202e-24 | L | 6.875738e-09 |
|  |  | V | 4.648197e-04 |
|  |  | W | 3.563835e-44 |
|  |  | Y | 2.146192e-09 |

**Table S6. Mean AAindex difference for optimized proteins obtained with MGEM.** The control consisted in (uniformly) randomly selecting replacement amino acids for the same number of positions as the corresponding MGEM mutant (avoiding the leading Met). AAindex values were fetched from [https://www.genome.jp/aaindex](https://www.genome.jp/aaindex/) (release 9.1 2006).

| **AAindex** | **Mean mutant**  **AAindex difference %** | **Mean random control**  **AAindex difference %** | **Publication** |
| --- | --- | --- | --- |
| MITS020101 | -18.753839 | 7.789475 | [(Richardson and Richardson 1988)](https://paperpile.com/c/9fsz4a/HglE) |
| TANS770108 | -17.832835 | -0.200313 | [(Woese 1973)](https://paperpile.com/c/9fsz4a/J1qc) |
| RICJ880117 | -5.443304 | 0.464476 | [(Mitaku, Hirokawa, and Tsuji 2002)](https://paperpile.com/c/9fsz4a/wTVi) |
| TANS770102 | -5.388635 | 0.659881 | [(Tanaka and Scheraga 1977)](https://paperpile.com/c/9fsz4a/PvJ1) |
| WOEC730101 | -2.380900 | -0.645872 | [(Vasquez, Nemethy, and Scheraga 1983)](https://paperpile.com/c/9fsz4a/6RRq) |
| VASM830101 | -1.854750 | 0.595889 | [(Nakashima and Nishikawa 1992)](https://paperpile.com/c/9fsz4a/xKxB) |
| GEOR030103 | -0.957061 | 0.023577 | [(Richardson and Richardson 1988)](https://paperpile.com/c/9fsz4a/HglE) |
| NAKH920107 | 0.413022 | -2.036270 | [(George and Heringa 2002)](https://paperpile.com/c/9fsz4a/mVHA) |
| RICJ880104 | 2.637145 | 0.691204 | [(Tanaka and Scheraga 1977)](https://paperpile.com/c/9fsz4a/PvJ1) |
| WERD780103 | 8.963559 | -2.015681 | [(Wertz and Scheraga 1978)](https://paperpile.com/c/9fsz4a/IDe9) |

**Table S7. Mean cost difference for optimized proteins obtained with MGEM.** The control consisted in (uniformly) randomly selecting replacement amino acids for the same number of positions as the corresponding MGEM mutant (avoiding the leading Met).

| **cost** | **Mean mutant**  **cost difference %** | **Mean random control**  **cost difference %** |
| --- | --- | --- |
| wagner_ferm | -13.603179 | 3.933833 |
| craig_steps | -13.306743 | 1.682922 |
| akashi | -9.218666 | 3.059934 |
| yeast_car_abs | -8.899055 | 1.926197 |
| wagner_resp | -8.736643 | 2.193350 |
| weight | -6.159937 | 0.863105 |
| craig_energy | -5.551885 | 3.734348 |
| yeast_nit_abs | -2.709100 | 1.179117 |
| yeast_car_rel | 1.978379 | -2.667477 |
| yeast_nit_rel | 11.187093 | -2.451302 |

**Table S8.** **The architecture of the BERT model with best performance.** The implementation was the TAPE *ProteinBertForValuePrediction* class, consisting of positional encoding, an encoder made up of multi-headed attention layers, and instead of a decoder (as in typical Transformer architectures), a thin (2 dense layers) multi-layer perceptron (MLP) predictor to a real value. To process our protein data, we implemented a specific TAPE learning task class to be used with the model. The network has 86.6 million trainable parameters.

| **BERT schematic** | **Network element** | **Value** |
| --- | --- | --- |
| 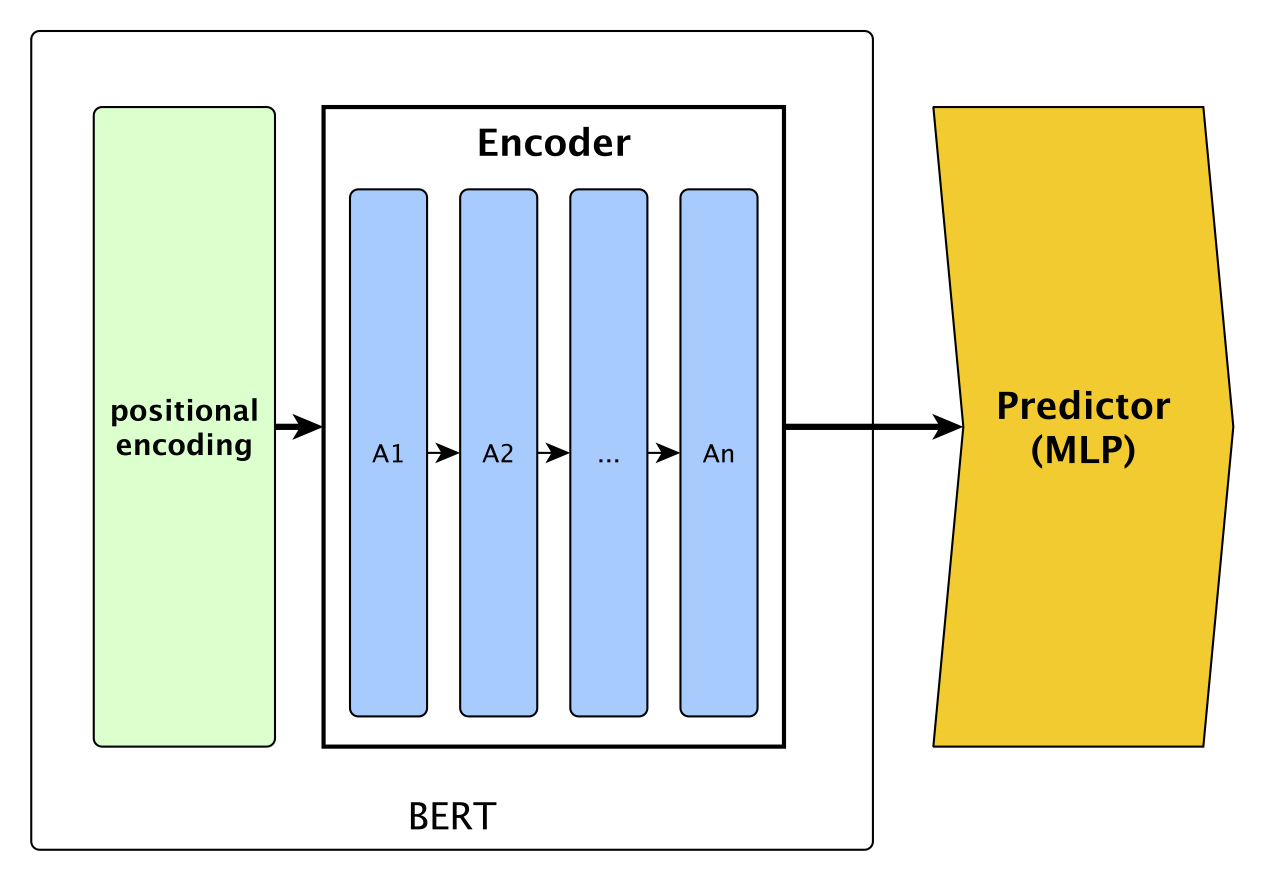 | vocab_size | 30 |
|  | type_vocab_size | 2 |
|  | max_position_embeddings | 1024 |
|  | num_hidden_layers (attention layers) | 8 |
|  | num_attention_heads | 4 |
|  | hidden_size (encoder embedding dim.) | 1024 |
|  | hidden_dropout_prob | 0.0 |
|  | attention_probs_dropout_prob | 0.0 |
|  | hidden_act | relu |
|  | initializer_range | 0.02 |
|  | layer_norm_eps | 1e-12 |
|  | intermediate_size (MLP) | 3072 |

**Table S9. BERT Training parameter values.** These were used for retraining the best model found by the hyperparameter search.

| **Training Parameter** | **Value** |
| --- | --- |
| learning_rate | 2.5681e-07 |
| batch_size | 32 |
| gradient_accumulation_steps | 16 |
| num_train_epochs | 500 |
| patience | 50 |

**Table S10. Hyperparameter search space for Transformer models.** The hyperparameter search was performed with Ray Tune using the *HyperBandForBOHB* scheduler (HyperBand that enables the BOHB Algorithm). Dropout was effectively disabled by fixing the hyperparameter range to zero. The Ray hyperparameter search used 10 samples per trial (choice of hyperparameter values).

| **Hyperparameter** | **Search space** |
| --- | --- |
| learning range | [1e-8, 1e-6] (uniform log-space sampling) |
| number of training epochs | {500, 800} |
| batch size | {16, 32, 64} |
| number of hidden layers | {8, 10, 12, 14} |
| number of attention heads | {4, 8, 16} |
| hidden size | {512, 1024} |
| intermediate size | {2048, 3072, 5120} |
| hidden dropout probability | 0 |
| attention dropout probability | 0 |
| hidden activation function | {gelu, relu} |

**Table S11.** List of PCR primers.

| **Primer name** | **Sequence** |
| --- | --- |
| pFA6-KanMX 488-507 FWD | GCAGTGAAAGATAAATGATCGCCGCGATTAAATTCCAACAGTTTTAGAGCTAGAAATAGC |
| pFA6-KanMX 488-507 REV | GCTATTTCTAGCTCTAAAACTGTTGGAATTTAATCGCGGCGATCATTTATCTTTCACTGC |
| pML_F | ACGCGCCCTGTAGCGGCGCA |
| f1 ori_R | TGCGCCGCTACAGGGCGCGT |
| M13R | CAGGAAACAGCTATGACC |
| YLR095C_F | GACACGACTAAGAAACTAGATCAATTGCTC |
| YLR095C_R | TACGGATGTGCGGCTGGAAAAGAAAG |

#

# Supplementary References

[Akashi, Hiroshi, and Takashi Gojobori. 2002. “Metabolic Efficiency and Amino Acid Composition in the Proteomes of Escherichia Coli and Bacillus Subtilis.” *Proceedings of the National Academy of Sciences of the United States of America* 99 (6): 3695–3700.](http://paperpile.com/b/9fsz4a/oO51)

[Barton, Michael D., Daniela Delneri, Stephen G. Oliver, Magnus Rattray, and Casey M. Bergman. 2010. “Evolutionary Systems Biology of Amino Acid Biosynthetic Cost in Yeast.” *PloS One* 5 (8): e11935.](http://paperpile.com/b/9fsz4a/neRHX)

[Craig, C. L., and R. S. Weber. 1998. “Selection Costs of Amino Acid Substitutions in ColE1 and ColIa Gene Clusters Harbored by Escherichia Coli.” *Molecular Biology and Evolution* 15 (6): 774–76.](http://paperpile.com/b/9fsz4a/qAZt)

[George, Richard A., and Jaap Heringa. 2002. “An Analysis of Protein Domain Linkers: Their Classification and Role in Protein Folding.” *Protein Engineering* 15 (11): 871–79.](http://paperpile.com/b/9fsz4a/mVHA)

[Ho, Brandon, Anastasia Baryshnikova, and Grant W. Brown. 2018. “Unification of Protein Abundance Datasets Yields a Quantitative Saccharomyces Cerevisiae Proteome.” *Cell Systems* 6 (2): 192–205.e3.](http://paperpile.com/b/9fsz4a/8pNWk)

[Li, Gang, Filip Buric, Jan Zrimec, Sandra Viknander, Jens Nielsen, Aleksej Zelezniak, and Martin K. M. Engqvist. 2022. “Learning Deep Representations of Enzyme Thermal Adaptation.” *Protein Science: A Publication of the Protein Society* 31 (12): e4480.](http://paperpile.com/b/9fsz4a/09Pm)

[Mitaku, Shigeki, Takatsugu Hirokawa, and Toshiyuki Tsuji. 2002. “Amphiphilicity Index of Polar Amino Acids as an Aid in the Characterization of Amino Acid Preference at Membrane–water Interfaces.” *Bioinformatics*  18 (4): 608–16.](http://paperpile.com/b/9fsz4a/wTVi)

[Nakashima, H., and K. Nishikawa. 1992. “The Amino Acid Composition Is Different between the Cytoplasmic and Extracellular Sides in Membrane Proteins.” *FEBS Letters* 303 (2-3): 141–46.](http://paperpile.com/b/9fsz4a/xKxB)

[Richardson, J. S., and D. C. Richardson. 1988. “Amino Acid Preferences for Specific Locations at the Ends of Alpha Helices.” *Science* 240 (4859): 1648–52.](http://paperpile.com/b/9fsz4a/HglE)

[Seligmann, Hervé. 2003. “Cost-Minimization of Amino Acid Usage.” *Journal of Molecular Evolution* 56 (2): 151–61.](http://paperpile.com/b/9fsz4a/5mGj)

[Tanaka, S., and H. A. Scheraga. 1977. “Statistical Mechanical Treatment of Protein Conformation. 5. A Multistate Model for Specific-Sequence Copolymers of Amino Acids.” *Macromolecules* 10 (1): 9–20.](http://paperpile.com/b/9fsz4a/PvJ1)

[Vasquez, Max, George Nemethy, and Harold A. Scheraga. 1983. “Computed Conformational States of the 20 Naturally Occurring Amino Acid Residues and of the Prototype Residue α-Aminobutyric Acid.” *Macromolecules* 16 (7): 1043–49.](http://paperpile.com/b/9fsz4a/6RRq)

[Wagner, Andreas. 2005. “Energy Constraints on the Evolution of Gene Expression.” *Molecular Biology and Evolution* 22 (6): 1365–74.](http://paperpile.com/b/9fsz4a/d9jW)

[Wertz, D. H., and H. A. Scheraga. 1978. “Influence of Water on Protein Structure. An Analysis of the Preferences of Amino Acid Residues for the inside or Outside and for Specific Conformations in a Protein Molecule.” *Macromolecules* 11 (1): 9–15.](http://paperpile.com/b/9fsz4a/IDe9)

[Woese, C. R. 1973. “Evolution of the Genetic Code.” *Die Naturwissenschaften* 60 (10): 447–59.](http://paperpile.com/b/9fsz4a/J1qc)
